# Antimicrobial Activity of Ibuprofen against Cystic Fibrosis-Associated Gram-Negative Pathogens

**DOI:** 10.1128/AAC.01574-17

**Published:** 2018-02-23

**Authors:** Parth N. Shah, Kimberly R. Marshall-Batty, Justin A. Smolen, Jasur A. Tagaev, Qingquan Chen, Christopher A. Rodesney, Henry H. Le, Vernita D. Gordon, David E. Greenberg, Carolyn L. Cannon

**Affiliations:** a Department of Microbial Pathogenesis and Immunology, Texas A&M University Health Science Center, College Station, Texas, USA; b Department of Pediatrics, University of Texas Southwestern Medical Center, Dallas, Texas, USA; c Department of Internal Medicine and Microbiology, University of Texas Southwestern Medical Center, Dallas, Texas, USA; d Center for Nonlinear Dynamics and Department of Physics, The University of Texas at Austin, Austin, Texas, USA

**Keywords:** Burkholderia spp., Pseudomonas aeruginosa, antimicrobial, cystic fibrosis, ibuprofen, nanoparticles

## Abstract

Clinical trials have demonstrated the benefits of ibuprofen therapy in cystic fibrosis (CF) patients, an effect that is currently attributed to ibuprofen's anti-inflammatory properties. Yet, a few previous reports demonstrated an antimicrobial activity of ibuprofen as well, although none investigated its direct effects on the pathogens found in the CF lung, which is the focus of this work. Determination of ibuprofen's *in vitro* antimicrobial activity against Pseudomonas aeruginosa and Burkholderia species strains through measurements of the endpoint number of CFU and growth kinetics showed that ibuprofen reduced the growth rate and bacterial burden of the tested strains in a dose-dependent fashion. In an *in vitro* Pseudomonas biofilm model, a reduction in the rate of biomass accumulation over 8 h of growth with ibuprofen treatment was observed. Next, an acute Pseudomonas pneumonia model was used to test this antimicrobial activity after the oral delivery of ibuprofen. Following intranasal inoculation, ibuprofen-treated mice exhibited lower CFU counts and improved survival compared with the control animals. Preliminary biodistribution studies performed after the delivery of ibuprofen to mice by aerosol demonstrated a rapid accumulation of ibuprofen in serum and minimum retention in lung tissue and bronchoalveolar lavage fluid. Therefore, ibuprofen-encapsulated polymeric nanoparticles (Ibu-NPs) were formulated to improve the pharmacokinetic profile. Ibu-NPs formulated for aerosol delivery inhibited the growth of *P. aeruginosa in vitro* and may provide a convenient dosing method. These results provide an additional explanation for the previously observed therapeutic effects of ibuprofen in CF patients and further strengthen the argument for its use by these patients.

## INTRODUCTION

Chronic infection and inflammation are the hallmarks of cystic fibrosis (CF) lung disease and are responsible for the majority of cases of morbidity and mortality in CF patients (1–3). Infections are polymicrobial, with typical pathogens including Staphylococcus aureus, Pseudomonas aeruginosa, Haemophilus influenzae, Burkholderia spp., Stenotrophomonas maltophilia, and Achromobacter xylosoxidans. The infections elicit an exuberant, acute inflammatory response dominated by neutrophils (4). In the distinctly altered microenvironment of the CF lung, the inflammation fails to clear the infection, causing the lung disease to progress through a self-perpetuating cycle of airway obstruction, chronic endobronchial infection, and excessive airway inflammation (4, 5), which eventually results in bronchiectasis and death (1, 2). Historically, alleviating the pulmonary obstruction and bacterial infection has been the mainstay of CF therapy; however, the recent recognition of inflammation as a primary cause of lung destruction has motivated the investigation of therapies directed against the excessive inflammatory response (4, 6, 7). For instance, oral and inhaled corticosteroids, macrolides, as well as nonsteroidal anti-inflammatory drugs (NSAIDs), such as ibuprofen, have been explored in several *in vivo* models and clinical trials and have shown an appreciable clinical effect (8–13).

Ibuprofen has appeared to be particularly advantageous due to its ability to target multiple inflammation pathways and its acceptable safety profile. In both a mouse model of acute Pseudomonas pulmonary infection (14) and a rat model of endotoxin-induced alveolitis (15), ibuprofen reduced the recruitment of neutrophils into the airways. Similarly, oral ibuprofen treatment in a rat model of chronic Pseudomonas endobronchial infection, resulting in a drug plasma concentration of 55 ± 24 μg/ml, led to a significant reduction in the inflammatory response and improved weight gain compared with placebo treatment (16). At this concentration, ibuprofen significantly reduced the level of leukotriene B_4_ production by stimulated rat neutrophil-rich leukocyte preparations; however, no effect on the pulmonary bacterial burden was observed (16). Thus, the beneficial effects of ibuprofen were attributed to inhibition of the lipoxygenase pathway and its downstream effects on neutrophils.

Subsequently, the safety and efficacy of high-dose ibuprofen (50- to 100-μg/ml plasma concentration) were evaluated in CF patients through clinical trials. In a randomized, double-blind, placebo-controlled trial conducted by Konstan et al., high-dose ibuprofen reduced the rate of decline of pulmonary function in patients with cystic fibrosis (CF) with good to excellent pulmonary function (10). A follow-up study analyzed data obtained from the CF registry for patients (age, 6 to 17 years) with a baseline forced expiratory volume in 1 s (FEV_1_) of >60% and demonstrated a significantly lower rate of decline of the FEV_1_ percentage predicted for patients treated with high-dose ibuprofen than for patients not treated with ibuprofen (17). Lands et al. have also investigated the safety and efficacy of high-dose ibuprofen in children (age, 6 to 18 years) with CF in a randomized, multicenter, double-blinded, placebo-controlled trial (12). Despite the absence of a statistically significant difference in the mean annual rate of decline in FEV_1_, a significant decrease in the annual rate of decline of the forced vital capacity (FVC) percentage predicted was observed between the ibuprofen and placebo groups. Collectively, these clinical trials document the benefits and relative safety of the long-term use of high-dose ibuprofen in CF patients and attribute these findings to the neutrophil-modulating properties of ibuprofen (7, 10, 12, 14, 15, 18–22).

Interestingly, a few reports in the literature have documented the antibacterial (23–29) and antifungal (23, 30) activity of ibuprofen, as well as its synergy with other antimicrobial agents both *in vitro* and *in vivo* (29–34). However, the direct effect of ibuprofen on bacterial pathogens prevalent in the CF lung has not yet been investigated in detail. Therefore, the studies described here aimed to probe the effects of high-dose ibuprofen (50 to 100 μg/ml) on strains of two important Gram-negative bacterial pathogens responsible for chronic pulmonary infections in CF patients, Pseudomonas aeruginosa and Burkholderia spp., through *in vitro* and *in vivo* studies. P. aeruginosa was chosen due to its prevalence in many CF patients and its ability to form biofilms, develop resistance, and develop a hypermutable phenotype, all of which contribute to poor survival outcomes (35–39). On the other hand, while few CF patients harbor Burkholderia spp., colonization by this pathogen poses a life-threatening problem because it is inherently antibiotic resistant (40). The results demonstrate the dose-dependent antimicrobial activity of ibuprofen *in vitro* against laboratory and clinical strains of both P. aeruginosa and Burkholderia spp. Furthermore, oral administration of ibuprofen thrice daily in a mouse model of acute lung infection resulted in a reduced bacterial burden, improved clinical illness scores, and superior survival compared with those achieved after sham treatment. Finally, since the lung is the target organ for therapy, the delivery of ibuprofen into healthy mice by aerosol was investigated, but the drug was rapidly transported from the lungs into the blood serum. As a solution to this pharmacokinetic problem, aerosolizable polymeric nanoparticle (NP) formulations of ibuprofen were developed, and their antimicrobial activity was demonstrated *in vitro*.

(Sections of this work were previously presented as a poster and a press release at the 115th General Meeting of the American Society for Microbiology, New Orleans, LA.)

## RESULTS

### Ibuprofen treatment reduces the bacterial burden in a dose-dependent fashion, and repeated ibuprofen treatment further extends this effect.

A dose-dependent reduction in the bacterial burden over 12 h was observed following ibuprofen treatment (50, 75, and 100 μg/ml) for each of the strains of Pseudomonas aeruginosa, Burkholderia cepacia, Burkholderia cenocepacia, and Burkholderia multivorans tested (Table 1). For instance, in the case of Pseudomonas aeruginosa strain PAO1, exposure to 50 μg/ml of ibuprofen over 12 h led to an approximately 0.5-log_10_ reduction in the bacterial count, which further increased to approximately 1 log_10_ and 1.5 log_10_ following exposure to 75 and 100 μg/ml ibuprofen, respectively. Exposure of other strains of P. aeruginosa to ibuprofen also resulted in a similar response, where the most significant reduction compared with the count obtained with the control treatment was observed at doses of 75 and 100 μg/ml. Of all strains, the greatest reduction in bacterial burden was observed for P. aeruginosa H25815 at an ibuprofen dose of 100 μg/ml, leading to an approximately 2-log_10_ reduction. Similarly, in the case of Burkholderia cenocepacia K56-2, exposure to 50 μg/ml of ibuprofen over 12 h led to an approximately 1/3-log_10_ reduction in the bacterial count, which further decreased by approximately 1 and 2 logs following exposure to 75 μg/ml and 100 μg/ml ibuprofen, respectively. These trends were conserved for other strains of Burkholderia spp. as well; the greatest dose-dependent reduction was observed for B. cenocepacia HI4277 (an ∼2.5-log_10_ reduction for 100 μg/ml). Separately, the possible inhibitory effect of dimethyl sulfoxide (DMSO) on bacterial growth was examined by growing the bacteria in Mueller-Hinton (MH) broth in the absence and presence of DMSO (2.5%, vol/vol), and as expected, no apparent differences between the two treatments were observed (data not shown).

**TABLE 1:** Antimicrobial activity in the presence and absence of ibuprofen over 12 h

Isolate	No. of log CFU/ml after treatment with IBU at the following concn (μg/ml)^*a*^:			
	0	50	75	100
Pseudomonas aeruginosa PAO1	2.69E+09	7.63E+08*	2.87E+08*	1.08E+08*
Pseudomonas aeruginosa M57-15	4.38E+09	2.27E+09	6.37E+08*	2.05E+08*
Pseudomonas aeruginosa T63547	7.91E+09	3.84E+09	5.59E+08*	2.70E+08*
Pseudomonas aeruginosa H25815	1.93E+09	1.57E+09	3.85E+08*	3.26E+07*
Burkholderia cenocepacia K56-2	1.24E+07	7.06E+06*	3.40E+06*	1.30E+05*
Burkholderia multivorans SH-2	7.18E+07	4.32E+07*	8.71E+06*	7.94E+05*
Burkholderia cepacia 1753	3.98E+07	1.61E+07*	6.28E+06*	6.74E+05*
Burkholderia cenocepacia HI4277	9.52E+06	6.46E+06	5.47E+05*	4.95E+04*

a Controls for the experiment include uninoculated MH broth and MH broth with DMSO (5%, vol/vol) without ibuprofen (IBU; 0 μg/ml). The mean numbers of log CFU are from at least three replicate experiments performed in duplicate wells (at least 6 sample replicates per treatment and pathogen) and were analyzed using a two-tailed paired *t* test. *, significant differences compared to the control (*P* ≤ 0.05).

**FIG 1:**
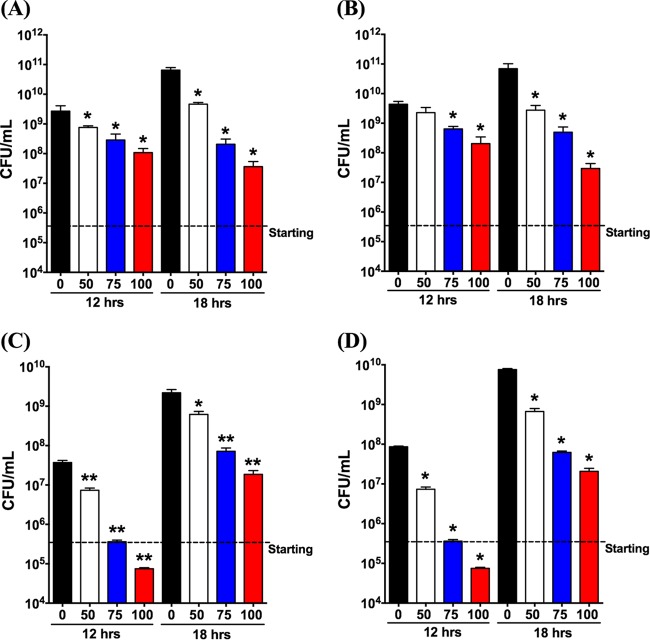
Additional ibuprofen treatment can extend the growth inhibition of Gram-negative bacteria. Pseudomonas aeruginosa (PAO1) (A), Pseudomonas aeruginosa (M57-15) (B), Burkholderia cenocepacia (K56-2) (C), and Burkholderia multivorans (D) were grown in the presence of 0, 50, 75, and 100 μg/ml of ibuprofen. Controls included uninoculated MH broth and MH broth containing DMSO (5%, vol/vol) without ibuprofen (0 μg/ml). At the 12-h time point, each sample was spiked with the appropriate treatment, and volumetric adjustments were performed to keep the concentrations constant. Each column represents the mean ± standard error of the mean of the number of CFU from at least 4 independent experiments with a total of at least 8 replicate samples per treatment. *P* values were determined by a two-tailed nonparametric Mann-Whitney test. *, *P* < 0.05 compared to the control; **, *P* < 0.01 compared to the control.

Next, the effects of an additional dose of ibuprofen applied at 12 h were investigated. Four of the previously tested strains (P. aeruginosa PAO1, P. aeruginosa M57-15, B. cenocepacia K56-2, and Burkholderia multivorans SH-2) were studied, and the CFU counts obtained under each condition were determined at 12 and 18 h. Regardless of the species, all cultures had CFU counts lower than those for the control at the 12-h time point. Growth inhibition was significantly influenced by the ibuprofen concentration and exhibited a dose-dependent decrease with an increase in the ibuprofen concentration (Fig. 1). Similar to the observations in Table 1, the effect of ibuprofen on P. aeruginosa PAO1 and M57-15 at 12 h was an approximately 1-log_10_ reduction of each strain at the highest dose tested (100 μg/ml) (Fig. 1A and B). After the additional spike and 6-h incubation, ibuprofen continued to impede bacterial growth compared with the control treatment. At the highest dose, further growth inhibition was observed for both P. aeruginosa PAO1 and M57-15, leading to bacterial burdens lower than those at 12 h. A more robust initial dose-dependent reduction in the bacterial burden was observed for both B. cenocepacia K56-2 and Burkholderia multivorans SH-2; the bacterial burden was approximately 3 log_10_ lower than that with the control treatment at 12 h (Fig. 1C and D). However, the initial decrease did not appear to be extended by additional spiking in either B. cenocepacia K56-2 or Burkholderia multivorans.

**FIG 2:**
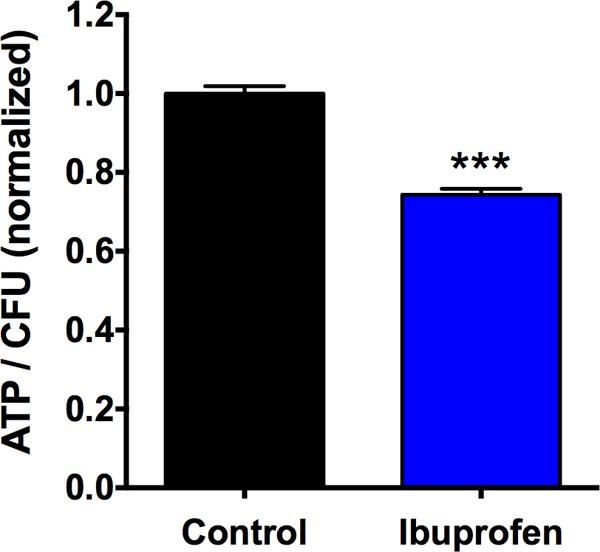
Ibuprofen exposure (100 μg/ml) leads to a reduction in intracellular ATP concentration in P. aeruginosa strain PAO1 compared with that in the untreated control bacteria (cultured in MH broth with DMSO and no ibuprofen). Data are shown as the mean ± standard error from 8 replicate experiments and were analyzed using a two-tailed, nonparametric Mann-Whitney test. ***, statistical significance with *P* < 0.001.

A few molecular mechanisms have been proposed to explain the antimicrobial activity of NSAIDs, including ibuprofen (30, 41–46). On the basis of the evidence regarding the ability of NSAIDs to act as a protonophore, the effects of ibuprofen exposure on ATP production in bacteria were explored as a first step. The preliminary observation, documented in Fig. 2, shows a significant reduction in the ATP content within Pseudomonas aeruginosa bacteria treated with ibuprofen (100 μg/ml, 30-min exposure) compared with that within the untreated control bacteria (*P* < 0.001). In contrast, bacteria treated with the sodium salt of ibuprofen (NaIbu) did not exhibit a significant reduction in the ATP content compared with that in the untreated controls (data not shown). The sodium salt of ibuprofen is an FDA-approved formulation found to have a more rapid onset of analgesic action than the protonated form of ibuprofen (47). The lack of antimicrobial activity of NaIbu supports the protonophore hypothesis, because this formulation is more polar and, hence, less likely to intercalate into the bacterial cell membrane.

### Ibuprofen delays the growth of bacteria in both standard medium and ASM.

**FIG 3:**
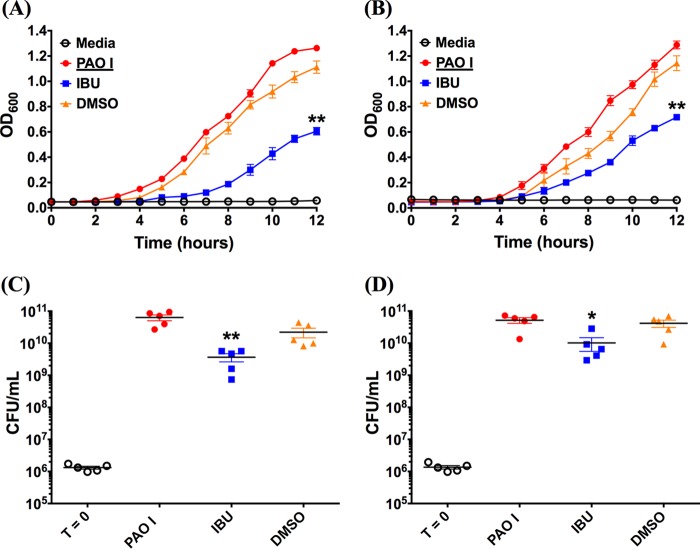
Ibuprofen reduces the growth rate and endpoint CFU counts of the Gram-negative bacterium PAO1 cultured in rich medium and artificial CF sputum (ASM). For both experiments, 1 × 10^6^ CFU/ml of PAO1 was grown in duplicate wells of a 96-well plate at 37°C in MH broth and artificial CF sputum in the presence or absence of ibuprofen (IBU; 100 μg/ml) over a 12-h period. The OD_600_ was determined at 1-h intervals to compare the growth rates for PAO1 in MH broth (A) and artificial CF sputum (B). At 12 h, the bacteria were diluted and plated to determine the bacterial burden (the number of CFU per milliliter), as shown, in MH broth (C) and artificial CF sputum (D). Controls included uninoculated growth medium (not shown), growth medium with bacteria alone, and growth medium containing DMSO (5%, vol/vol) without ibuprofen (0 μg/ml) inoculated with bacteria. Each data set represents the mean from 5 independent experiments (total, 10 replicates per treatment), and the data are presented as the mean ± standard error. Symbols in panels C and D: empty circles, starting inoculum of bacteria (T = 0, time zero); filled circles, bacteria alone; filled squares, bacteria treated with ibuprofen; filled upright triangles, bacteria in growth medium containing DMSO but no ibuprofen. A paired *t* test was used to analyze the differences in the endpoint OD_600_ for growth curves, and a two-tailed, nonparametric Mann-Whitney test was used to analyze the differences in CFU counts. *, *P* < 0.05 compared to the control; **, *P* < 0.01 compared to the control.

**FIG 4:**
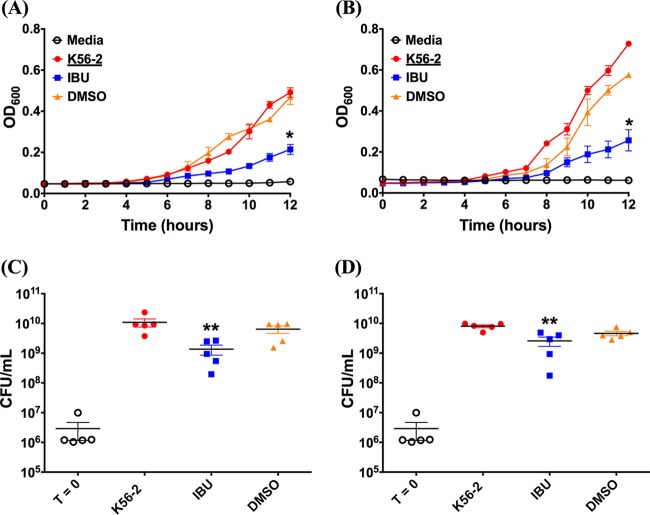
Ibuprofen reduces the growth rate and the endpoint CFU counts of the Gram-negative bacterium B. cenocepacia K56-2 cultured in rich medium and artificial CF sputum (ASM). For both experiments, 1 × 10^6^ CFU/ml of B. cenocepacia K56-2 was grown in duplicate wells of a 96-well plate at 37°C in MH broth and artificial CF sputum in the presence or absence of ibuprofen (IBU; 100 μg/ml) over a 12-h period. The OD_600_ was determined at 1-h intervals to compare the growth rates for B. cenocepacia K56-2 in MH broth (A) and artificial CF sputum (B). At 12 h, the bacteria were diluted and plated to determine the bacterial burden (the number of CFU per milliliter), as shown, in MH broth (C) and artificial CF sputum (D). Controls included uninoculated growth medium (not shown), growth medium with bacteria alone, and growth medium containing DMSO (5%, vol/vol) without ibuprofen (0 μg/ml) inoculated with bacteria. Each data set represents the mean from 5 independent experiments (total, 10 replicates per treatment), and the data are presented as the mean ± standard error. Symbols in panels C and D: empty circles, starting inoculum of bacteria; filled circles, bacteria alone; filled squares, bacteria treated with ibuprofen; filled upright triangles, bacteria in growth medium containing DMSO but no ibuprofen. A paired *t* test was used to analyze the differences in the endpoint OD_600_ for growth curves, and a two-tailed, nonparametric Mann-Whitney test was used to analyze the differences in CFU counts. *, *P* < 0.05 compared to the control; **, *P* < 0.01 compared to the control.

Since bacterial growth characteristics are a function of the growth medium, we compared the growth rate and the endpoint number of CFU of two representative Gram-negative pathogen strains (P. aeruginosa PAO1 and B. cenocepacia K56-2) in MH broth and artificial sputum medium (ASM). ASM has been designed to mimic the sputum of CF patients and provides nutritional conditions similar to those of CF sputum, as well as synthetic CF sputum medium (48). Further, ASM has gained wide acceptance for use in the development of assays requiring conditions that simulate the CF lung (49–52). When starting with a similar inoculum (1 × 10^6^ CFU/ml), the growth curves for both PAO1 and B. cenocepacia K56-2 in MH broth (Fig. 3A and 4A) and ASM (Fig. 3B and 4B) resembled a typical exponential-type growth curve for bacteria; however, a slightly longer but not significantly different lag time was observed for the ibuprofen treatment compared with that for the control treatments consisting of medium only and medium containing DMSO. Furthermore, in each instance, the slopes of the growth curves suggest that bacteria subjected to ibuprofen treatment grew at a lower rate than the control bacteria. Consequently, the final values of the optical density at 600 nm (OD_600_) for ibuprofen-treated bacteria were significantly lower than the values of the OD_600_ for control bacteria at 12 h in both MH broth and ASM (*P* < 0.01 for PAO1 and *P* < 0.05 for B. cenocepacia K56-2). As expected, no differences in the growth rates or final OD_600_ values were observed for bacteria grown in the control treatments consisting of untreated medium only and medium containing DMSO, irrespective of the choice of medium, suggesting that the presence of DMSO does not hamper bacterial growth over 12 h. Extending the time for the growth of PAO1 in MH broth to 24 h continued to demonstrate the difference between ibuprofen in DMSO and the controls consisting of MH alone or MH plus DMSO (*P* < 0.0001 and *P* < 0.05, respectively; see Fig. S1 in the supplemental material). Separately, the growth of B. cenocepacia K56-2 with the two control treatments was faster in ASM than in MH broth; however, the growth characteristics of the PAO1 control strain remained largely unchanged between the two media. The lower growth rate and lower final OD_600_ values of ibuprofen-treated bacteria in each instance also translated to a significant reduction in bacterial burdens compared with those after the control treatments at 12 h. In the case of PAO1, when MH broth was used (Fig. 3C), the bacterial burden following ibuprofen treatment was approximately 1.5 log_10_ lower than that following the control treatments (*P* < 0.01). This reduction in the number of CFU was somewhat diminished, yet it was significant (*P* < 0.05) when the medium was changed to ASM (Fig. 3D). Similar results were observed when the effect of ibuprofen on the endpoint number of CFU of B. cenocepacia K56-2 in both MH broth and ASM was investigated. An approximately 1-log_10_ reduction in the mean endpoint number of CFU of B. cenocepacia K56-2, which was significantly lower than that for the untreated controls (*P* < 0.01), was observed in MH broth (Fig. 4C). Again, this reduction in the number of CFU was somewhat diminished when ASM was used, yet the differences between the ibuprofen-treated bacteria and the untreated control bacteria were significant (*P* < 0.01) (Fig. 4D).

### Ibuprofen treatment delays the onset of biomass accumulation in Pseudomonas aeruginosa biofilms grown *in vitro*.

**FIG 5:**
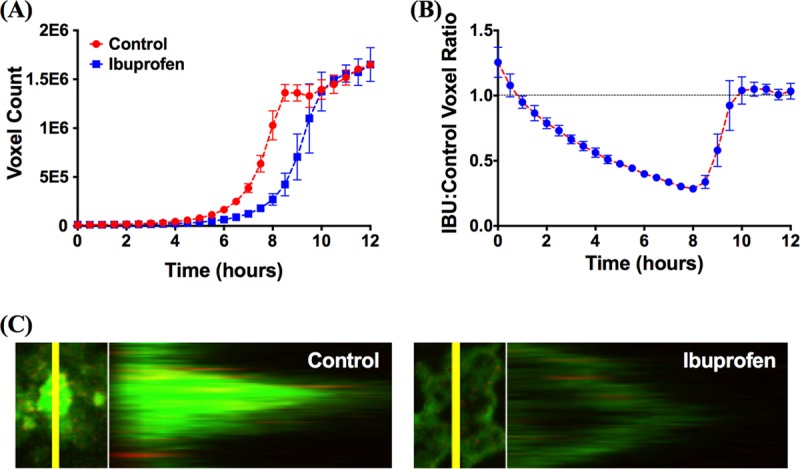
Ibuprofen at a concentration of 100 μg/ml increases the lag time before bacterial biofilms begin exponential growth. (A) Comparison of growth curves of untreated (control) biofilms and biofilms exposed to ibuprofen, demonstrating an increase in the lag time and a consequent reduction in the biomass accumulation of P. aeruginosa biofilms exposed to 100 μg/ml ibuprofen. (B) Ratios of ibuprofen-treated to untreated biofilm growth over time. All data are shown as the mean ± standard error of the mean from 3 replicate experiments performed in pairs on separate days (total, 6 replicates per treatment). A replicated, pairwise comparison of experiments done on the same day allowed us to normalize out for the effects of day-to-day experimental variation; we have used a similar approach for other biofilm studies (95). (C) Confocal and z-stack images of P. aeruginosa control biofilms or biofilms continuously exposed to 100 μg/ml ibuprofen.

Figure 5A shows a comparison of the level of biomass accumulation in untreated (control) biofilms and ibuprofen-treated (100 μg/ml) biofilms growing under similar conditions, where the voxel count served as a measure of biomass accumulation. For both untreated and ibuprofen-treated biofilms, the biomass accumulation over a 1.5- to 2-h window at the beginning of growth was subexponential. The subsequent transition to exponential growth occurred later for the ibuprofen-treated biofilms than for the control biofilms, and an initial subjective determination suggested that the delay was 0.5 ± 0.41 h (mean ± standard deviation). However, since this determination was limited to no better than half an hour in time resolution, further analysis of these data was performed by taking a second derivative with respect to the time of the voxel counts, and the original estimate of the delay for ibuprofen-treated biofilms was revised to 0.86 ± 0.27 h (mean ± standard deviation). Once the biofilms reached an exponential growth phase, the bacterial doubling time in ibuprofen-treated biofilms was 1.8 ± 0.29 h (mean ± standard deviation), which was not significantly different from the doubling time for the bacteria in the control biofilms (1.5 ± 0.11 h). Figure 5B documents another representation of this phenomenon, where the level of biomass accumulation in the ibuprofen-treated biofilms was normalized to that in the control biofilms. While the initial biomass in the ibuprofen-treated biofilms was slightly higher than that in the control biofilms, a sharp decline in the voxel count ratio (ibuprofen-treated biofilms/control biofilms) was observed almost immediately. This ratio continued to drop and reached its lowest value of 0.285 at 8 h, with the curve appearing similar to a curve for an exponential decay profile. Subsequently, the ratio began to rise almost linearly and reached a value of 1 at approximately 10 h. While this observation may suggest a comparable biomass for control and ibuprofen-treated biofilms by 10 h of growth, underestimation of the biomass of the ibuprofen-treated biofilms may occur when they become confluent, typically at 9 h. The thick ibuprofen-treated biofilms displayed large volumes that were dim or completely dark; this phenomenon may result from a low oxygen concentration deep within the biofilm, which reduces bacterial metabolism and impedes green fluorescent protein (GFP) production and folding (Fig. 5C). Examination under transmitted light confirmed that the biofilm was present in these dim or dark regions. However, the measurement of biomass counted only fluorescent regions. Thus, whether the biomass accumulation for both control and ibuprofen-treated biofilms after 9 h was truly equal or only appeared to be equal due to an imaging artifact is currently unknown and is a topic of further investigation.

### Oral delivery of ibuprofen achieves therapeutic concentrations in serum, reduces the bacterial burden, and improves survival in PAO1-infected mice.

**FIG 6:**
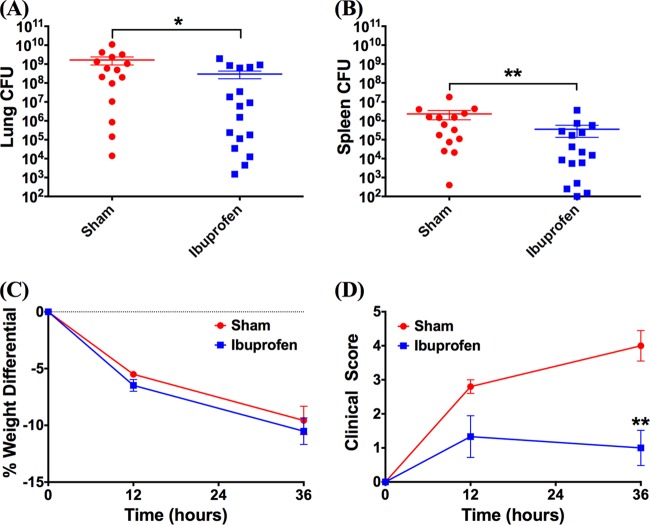
Ibuprofen reduces the bacterial burden in the lungs and spleens of PAO1-infected mice and improves the clinical scores at 36 h postinfection. For experiments for determination of the number of CFU, male C57BL/6J mice were intranasally inoculated with ∼5 × 10^5^ CFU P. aeruginosa (PAO1), orally treated with ibuprofen (*n* = 17) or vehicle (*n* = 15) at the designated time points, and sacrificed at 36 h. (A, B) Significant reductions in the bacterial burdens in the lung (A) and the spleen (B) were observed following ibuprofen treatment. (C, D) No significant differences in weight loss were observed between ibuprofen- and sham-treated mice (C), but ibuprofen treatment significantly reduced the clinical scores of mice at 36 h compared with sham treatment (D). Data are displayed as the mean ± standard error of the mean and were analyzed using a two-tailed, nonparametric Mann-Whitney test. In the case of the experiments determining the number of CFU, each symbol represents an individual mouse. *, statistical significance with *P* < 0.05; **, statistical significance with *P* < 0.01.

**FIG 7:**
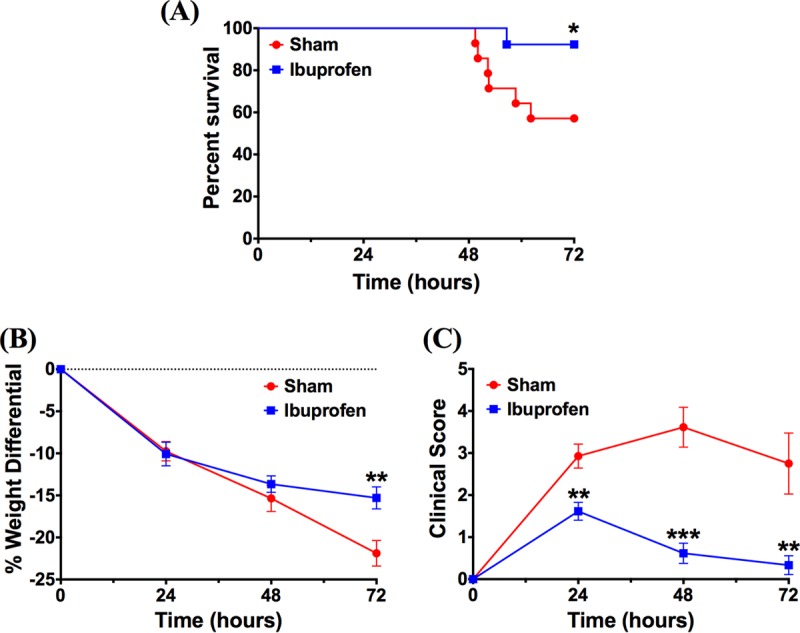
Ibuprofen treatment improves survival and reduces weight loss as well as clinical scores in PAO1-infected mice. Male C57BL/6J mice were intranasally inoculated with ∼5 × 10^5^ CFU P. aeruginosa (PAO1), orally treated with ibuprofen (*n* = 13) or the vehicle (*n* = 14) at the designated time points, and evaluated for 72 h. (A) Kaplan-Meier survival curves demonstrate greater survival in ibuprofen-treated mice than sham-treated mice (92.3% survival in ibuprofen-treated mice [12/13 mice] versus 57.1% in sham-treated mice [8/14 mice]; *P* = 0.0386, as analyzed using a log-rank Mantel-Cox test). (B, C) Ibuprofen treatment also reduced weight loss on day 3 (B) and improved the clinical scores of PAO1-infected mice at each time point (C). Data for weight loss and clinical scores are displayed as the mean ± standard error of the mean and were analyzed using a two-tailed, nonparametric Mann-Whitney test. *, statistical significance with *P* < 0.05; **, statistical significance with *P* < 0.01; ***, statistical significance with *P* < 0.001.

To test the *in vivo* antimicrobial activity of ibuprofen, a mouse model of acute P. aeruginosa pneumonia was used following preliminary dosing studies in healthy mice. Dosing experiments revealed that an oral dosage of 0.75 mg ibuprofen resulted in a blood serum concentration of 124.22 ± 15.40 μg/ml at 1 h posttreatment, whereas a negligible amount of ibuprofen was detected in the sham-treated animals (0.12 μg/ml). These results provide validation that the ibuprofen concentration necessary to achieve antimicrobial effects (>50 μg/ml, based on *in vitro* studies) can be realized in the serum of mice following oral delivery. Subsequently, as an initial evaluation of the antimicrobial activity of ibuprofen, the bacterial burdens in the lungs and spleens of mice infected with a 50% lethal dose (LD_50_) of P. aeruginosa strain PAO1 were compared. Following five treatment doses over 36 h postinfection, significant reductions in both lung (Fig. 6A) and spleen (Fig. 6B) bacterial burdens were observed for the mice treated with ibuprofen. At the 36-h time point, the sham-treated mice had approximately 1 log_10_ more bacteria in both their lungs (*P* = 0.0314) and spleens (*P* = 0.0096) than the ibuprofen-treated mice. A concurrent evaluation of other health-associated parameters, such as weight loss and clinical scores (Fig. 6C and D), showed no significant effect of ibuprofen treatment on weight loss after infection over 36 h (*P* = 0.6); however, at the same time point, the ibuprofen-treated mice exhibited clinical scores significantly reduced from those for the sham-treated animals (*P* = 0.002). During the survival study, the ibuprofen-treated mice exhibited less weight loss (*P* = 0.0095) than the sham-treated mice on day 3 (Fig. 7B), and the clinical scores of the ibuprofen-treated mice were significantly lower than those of the sham-treated mice on all days (day 1, *P* = 0.001; day 2, *P* = 0.0002; day 3, *P* = 0.0045) (Fig. 7C). Further, in these PAO1-infected mice, a significant survival advantage for the ibuprofen-treated mice (92%) compared with that for the sham-treated animals (57%) (*P* = 0.0386) was observed at 72 h (Fig. 7A).

### Delivery by nebulization results in the rapid transport of ibuprofen to serum, necessitating the use of controlled-delivery nanoparticle formulations.

**FIG 8:**
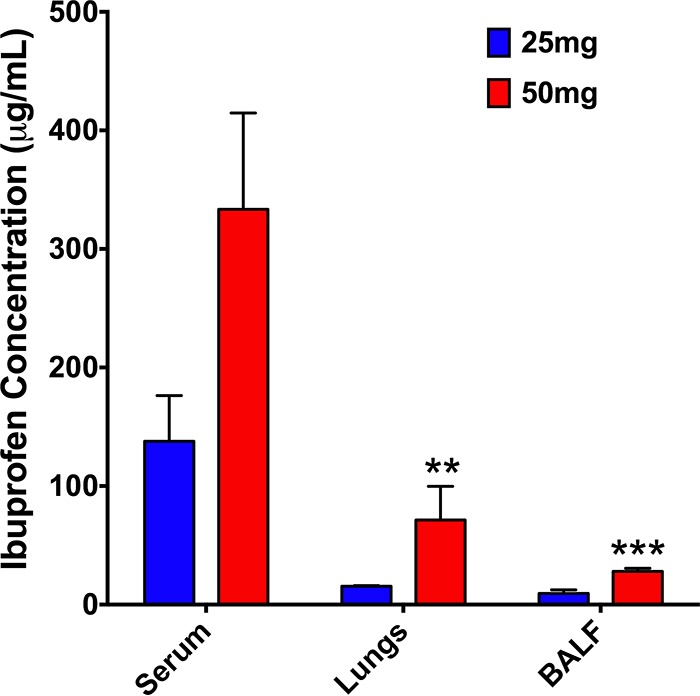
Biodistribution studies demonstrating the abundance of ibuprofen in the serum but low concentrations in the lungs and bronchoalveolar lavage fluid (BALF) of mice immediately after the delivery of 25 or 50 mg ibuprofen to healthy C57BL/6J mice by nebulization. Doses of 25 and 50 mg refer to the amount of ibuprofen placed in the Aeroneb micropump nebulizer (Aerogen Inc.). Data are presented as the mean ± standard error for 3 replicate mice per treatment, with each sample being analyzed in duplicate (total, 6 replicates per treatment), and were analyzed using two-way ANOVA with Tukey's multiple-comparison *post hoc* test. **, statistical significance with *P* < 0.01 compared to the serum ibuprofen concentration for a given dose; ***, statistical significance with *P* < 0.001 compared to the serum ibuprofen concentration for a given dose.

Next, the possibility of delivering ibuprofen to the lungs of healthy (uninfected) mice via nebulization was explored. Ibuprofen was solubilized in phosphate buffer containing 1% (vol/vol) DMSO. This formulation was nebulized using an Aeroneb lab apparatus, which is based on the vibrating mesh technology frequently used to deliver antibiotics to the lungs of CF patients. Analysis of ibuprofen concentrations in the blood serum, lung tissue, and bronchoalveolar lavage fluid (BALF) following nebulization of 25- and 50-mg doses demonstrated that the 50-mg dose led to a concentration of ibuprofen in each of the tissue or fluid samples approximately twice that achieved with the 25-mg dose, as expected (Fig. 8). Using the serum and tissue ibuprofen concentrations and a predicted blood volume of 1.5 ml for a 25-g mouse for calculations, approximately 0.8% of the 25-mg dose and 1.0% of the 50-mg dose were delivered to the mice via nebulization. However, the rapid transport of this small-molecule drug into the blood was observed, leading to serum concentrations that were 12 to 15 times higher than the BALF concentrations and 4 to 5 times higher than the lung tissue concentrations. Additionally, the nebulization times for these ibuprofen formulations were unreasonably long (∼43 min and 70 min for the 25- and 50-mg doses, respectively). Thus, the attempts to achieve concentrations of ibuprofen higher in the lungs than in the serum through aerosol delivery failed.

**FIG 9:**
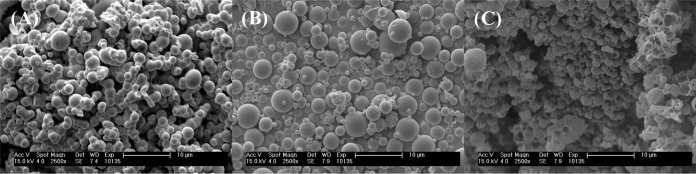
Scanning electron micrographs demonstrating the surface morphology and size distribution of PLGA (PLGA-PEG) nanoparticles formulated using an oil-in-water (o/w) emulsion technique. (A) Empty nanoparticles; (B) ibuprofen-loaded nanoparticles; (C) ibuprofen-loaded nanoparticles formulated with sodium ibuprofen in the external aqueous phase.

**FIG 10:**
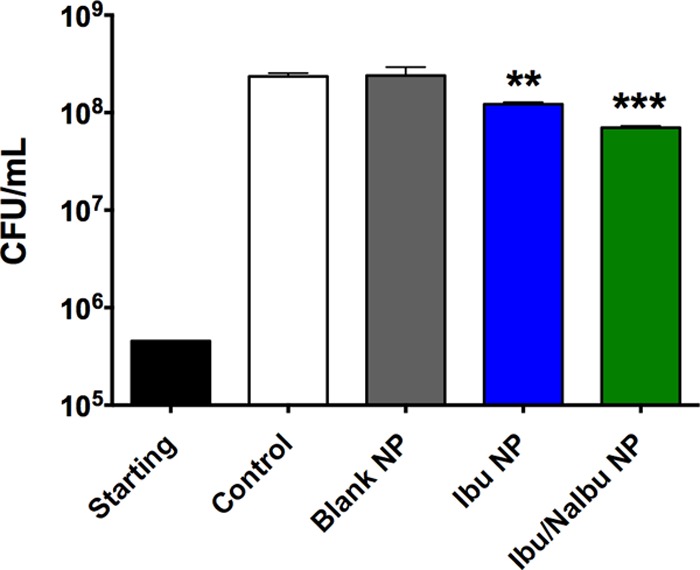
Nanoparticles encapsulating ibuprofen impede the growth of P. aeruginosa (PAO1) compared with the growth obtained with the controls and empty nanoparticles, as demonstrated by studies of the endpoint number of CFU over 6 h. Ibu/NaIbu-NPs were formulated using an oil-in-water emulsion technique with sodium ibuprofen in the external phase to increase ibuprofen loading within the nanoparticles. A total of 6 replicate samples (2 duplicates × 3 technical replicates) were assessed for each treatment. The data are presented as the mean ± standard error and were analyzed using one-way ANOVA with Tukey's multiple-comparison *post hoc* test. **, statistical significance with *P* < 0.01 compared to the control; ***, statistical significance with *P* < 0.001 compared to the control.

As an alternative, nebulizable nanoparticulate formulations encapsulating ibuprofen were prepared. Nanoparticles were prepared using a mixture of poly(lactide-co-glycolide) (PLGA) and a copolymer of PLGA and polyethylene glycol (PEG) (PLGA-PEG) and an oil-in-water (o/w) emulsion technique, wherein PLGA forms the core of the nanoparticles and PLGA-PEG primarily concentrates at the surface of the NPs. Scanning electron microscopy (SEM) was used to examine the surface morphology and size distribution of three nanoparticle formulations (Fig. 9). Both empty nanoparticles (Fig. 9A) and ibuprofen-loaded nanoparticles (Fig. 9B) were spherical and exhibited a smooth morphology with minimal defects on the particle surface. On the other hand, SEM images of ibuprofen-loaded nanoparticles formulated with sodium ibuprofen (NaIbu) in the external aqueous phase (Fig. 9C) showed a mixture of spherical particles which possessed either a smooth surface morphology or a rough surface morphology with significant surface defects in the form of pores or divots. Additionally, some incompletely formed or disrupted particles, as well as polymeric debris, were also observed (Fig. 9C). While minimal aggregation was observed for both formulations of ibuprofen-loaded nanoparticles, empty nanoparticles demonstrated a larger degree of aggregation. Some empty nanoparticles also appeared to fuse during the formulation process. In each instance, the particle size distribution appeared to be bimodal, with smaller particles exhibiting diameters of the order of a few hundred nanometers and larger particles exhibiting diameters in the micron range (up to ∼4 μm). Interestingly, the nanoparticles formulated with NaIbu in the external phase had the sharpest size distribution, with most particles being submicron sized in diameter. Ibuprofen loading studies revealed that addition of NaIbu to the external phase improved the loading of ibuprofen within the nanoparticles significantly. These particles contained 16% ibuprofen by weight (∼160% encapsulation efficiency), whereas the particles formulated without NaIbu in the external phase contained ∼4% ibuprofen by weight (∼40% encapsulation efficiency). Finally, preliminary investigations of the antimicrobial activity of ibuprofen-loaded nanoparticles using a study of the endpoint number of CFU were performed (Fig. 10). No differences in CFU counts were observed between bacteria treated with empty nanoparticles and those treated with the medium only as a control (*P* = 0.9998). In contrast, treatment with the ibuprofen-loaded nanoparticle formulations revealed a significant reduction in the CFU counts of P. aeruginosa strain PAO1 compared with the counts achieved with the control treatments consisting of empty nanoparticles and medium only (*P* < 0.001), with the highest reduction in bacterial burden being observed for NPs encapsulating ibuprofen with NaIbu in the external phase (Ibu/NaIbu-NPs) (∼0.6 log_10_).

## DISCUSSION

The use of ibuprofen for the treatment of chronic inflammation in cystic fibrosis (CF) lung disease has well-documented beneficial effects, which have been attributed to its ability to act on multiple inflammatory pathways (18–22, 53–56). Yet, a few reports in the literature also document the antimicrobial properties of ibuprofen ranging from bacteriostatic to bactericidal against several different pathogens, as well as its synergy with other antimicrobials (23–29). These reports are further augmented by additional observations of the antimicrobial and synergistic activities of other NSAIDs, such as salicylate, acetylsalicylic acid, indomethacin, sulindac, and diclofenac, which have been summarized in a recent minireview (57). Collectively, these data raise the possibility that the observed beneficial effects in CF lung disease may be due to a combination of its anti-inflammatory and antimicrobial activities rather than due to its anti-inflammatory activity alone. Because the direct antimicrobial effects of ibuprofen on CF-associated Gram-negative pathogens have not been previously investigated, we sought to systematically explore this effect in two important CF pathogens: Pseudomonas aeruginosa and Burkholderia spp. Further, our studies were conducted at ibuprofen concentrations ranging from 50 to 100 μg/ml, because doses in this range are clinically achievable and were previously shown to have a beneficial effect in CF patients (10).

Our initial screening studies with several strains of P. aeruginosa and Burkholderia spp. demonstrated a dose-dependent reduction in the bacterial burden for all strains at 12 h following exposure to ibuprofen (Table 1). At the highest ibuprofen dose of 100 μg/ml, the bacterial reduction for Burkholderia species strains was ∼2 log_10_ compared with the count for the controls, whereas for the P. aeruginosa strains the reduction was ∼1 to 1.5 log_10_. These results are in partial agreement with other data in the literature documenting the antimicrobial effects of ibuprofen ranging from bacteriostatic to bactericidal. For example, Sanyal et al. have demonstrated MICs of 40 to 80 μg/ml against a Staphylococcus aureus strain at pH 5 (23). Similarly, Elvers and Wright observed a concentration- and pH-dependent bacteriostatic effect of ibuprofen on S. aureus and Staphylococcus epidermidis. Growth suppression occurred at ibuprofen concentrations greater than 150 μg/ml at an initial pH of 7, and almost complete growth inhibition was observed at an ibuprofen concentration of 450 μg/ml (24). This effect was further enhanced at pH 6, at which almost complete growth inhibition occurred at an ibuprofen concentration of just 125 μg/ml (24). Separately, in the case of Helicobacter pylori, complete growth inhibition of three strains was observed at ibuprofen concentrations ranging from 50 to 125 μg/ml, and an increase in dose to 250 μg/ml or greater led to an onset of bactericidal activity (25). Additionally, recent findings by Guzman et al. document the complete growth inhibition of Mycobacterium tuberculosis H_37_Rv at an ibuprofen concentration of 75 μg/ml, while the MICs against M. bovis BCG, M. aurum, and M. neoaurum were 90, 65, and 65 μg/ml, respectively, using a high-throughput spot culture growth inhibition (HT-SPOTi) assay *in vitro* (28). These effects also extended to multidrug-resistant clinical strains 11:139, 11:169, and 11:368 of M. tuberculosis, with MICs ranging from 30 to 50 μg/ml (28). When correlated with the CFU counts, treatment of M. bovis BCG with ibuprofen at 180 μg/ml resulted in a ∼2-log_10_ reduction in the bacterial burden, provided that ibuprofen was introduced at the beginning of culture; however, introduction of ibuprofen at the beginning of exponential growth phase led to a minimal reduction in the number of CFU. Interestingly, no growth inhibition of bacteria such as Rhodococcus equi RHA1, Pseudomonas putida, Escherichia coli DH5α, and Staphylococcus aureus strains ATCC 25923 and EMRSA-16 was observed at an ibuprofen concentration of 100 μg/ml when using the HT-SPOTi assay (28). Furthermore, suppression of the growth of the Gram-negative pathogens Pseudomonas fluorescens and E. coli was not observed by Elvers and Wright even at an ibuprofen concentration of 350 μg/ml (24). These seemingly contradictory results, coupled with a lack of explanation regarding the selectivity of ibuprofen in these reports, lead us to believe that differences in the experimental protocols, such as the choice of assay used for the determination of antimicrobial activity, the ibuprofen formulation, growth conditions, and the choice of endpoint, play a considerable role in the observed outcomes. As a case in point, the HT-SPOTi assay was performed over 16 h; however, in the case of fast-growing bacterial pathogens in our studies, following the initial 8 to 12 h of exposure, we found that the ability of ibuprofen to suppress bacterial growth was substantially diminished.

Thus, we sought to explore whether redosing the bacterial cultures with ibuprofen at 12 h could prolong the observed growth suppression. Our rationale for performing these studies was driven by the available data on the formulation-dependent biological half-life of ibuprofen (1.3 to 3 h) *in vivo* (58), as well as its dosing at 12-h intervals both *in vivo* and in CF patients (10, 12, 16, 54). Furthermore, previous observations suggested that various microorganisms have the ability to utilize NSAIDs, such as ibuprofen, as substrates, resulting in their biodegradation, as well as biotransformation (59–62). Indeed, our results demonstrated that the concentration-dependent antimicrobial activity of ibuprofen was extended to 18 h following the introduction of fresh ibuprofen into the system at 12 h (Fig. 1). Interestingly, the initial growth suppression was more robust for the two Burkholderia species strains than for the two P. aeruginosa strains, yet at 18 h the results were more striking in the case of P. aeruginosa, for which an additional 0.5- to 1.0-log_10_ reduction in CFU counts (compared with the number of CFU at 12 h) was observed after the medium was spiked with the highest ibuprofen concentration. These data suggest a continued reduction in the growth rate and, subsequently, the P. aeruginosa burden after ibuprofen retreatment. In contrast, no distinct advantage of spiking with ibuprofen was evident in the case of the Burkholderia strains, except for the conservation of the trend pertaining to the dose-dependent lowering of the CFU counts compared with those from the control treatment. These results, coupled with the observation of Guzman et al. (28) that addition of ibuprofen at the start of exponential growth phase failed to achieve a reduction in the number of CFU, point toward interference with a metabolic pathway as being one of the targets for the antimicrobial mechanism of action of ibuprofen. Indeed, our preliminary foray in this area showed a significant depletion in the intracellular ATP concentration in PAO1 bacteria compared with that in untreated control bacteria (cultured in MH broth with DMSO) following exposure for 30 min (Fig. 2), suggesting that ibuprofen potentially uncouples oxidative phosphorylation in bacteria. Usually weakly acidic, hydrophobic (typically aromatic) compounds in their anionic state exhibit the highest potential to delocalize a negative charge, thus acting as uncouplers (43). Most commonly used NSAIDs have these characteristics, and studies have demonstrated that weakly acidic NSAIDs, including ibuprofen, cause uncoupling in mitochondria (41, 42). Note that the sodium salt of ibuprofen (NaIbu) is not a weak acid and, thus, as expected, did not demonstrate activity as an uncoupler. Interestingly, the presence of a free carboxylic acid moiety was deemed critical for the antitubercular activity of ibuprofen and its analogs (28), further validating our observation. However, the ability of ibuprofen to induce this disruption in energy generation might depend on the growth stage of the individual bacterial species, thereby possibly explaining the species-specific suppression effects that we observed in our spiking experiments. Interestingly, recent chemoinformatics- and bioinformatics-based studies by Kahlous et al. in combination with bioassays demonstrated structural similarities between ibuprofen and the quinolone and fluoroquinolone class of antimicrobials, such as ciprofloxacin, levofloxacin, and nalidixic acid (46). The authors further reported that ibuprofen and other NSAIDs have the ability to bind the protein target, i.e., DNA gyrase, of antibiotics to which they are similar and inhibit bacterial growth (46). Whether the reduction in ATP generation influences DNA gyrase-mediated antimicrobial activity or other important downstream cellular processes is currently unknown.

All of our experiments were performed using Mueller-Hinton broth, which is a general-purpose medium used for the culture of a wide variety of microorganisms and microbiological assays. However, the choice of growth medium can have significant effects on growth and viability, gene expression, and metabolic functions in microorganisms. Additionally, the growth conditions for bacterial pathogens in the CF lung are markedly different due to the presence of viscous mucus, comprising breakdown products of inflammatory and epithelial cells, including copolymers of DNA and filamentous (F) actin, bacteria, cell debris, and variable amounts of mucin (63, 64), which also serve as nutritional sources for these pathogens (65). For example, Palmer and coworkers have demonstrated significant differences in Pseudomonas aeruginosa physiology when the bacteria are grown in CF sputum and when they are grown in glucose (66). Therefore, we investigated the effect of the growth medium on ibuprofen's antimicrobial activity by incubating PAO1 and B. cenocepacia K56-2 in the presence and absence of ibuprofen in both MH broth and artificial sputum medium (ASM) (Fig. 3 and 4). A lower growth rate and a reduction in the endpoint OD_600_ (12 h) were observed for both bacteria in MH broth as well as ASM, which translated to an approximately 1-log_10_ reduction in CFU counts compared with those for the controls (untreated bacteria and bacteria cultured in MH with DMSO). Thus, these results suggest that the growth-suppressive antimicrobial activity of ibuprofen is conserved irrespective of the growth medium.

Next, given the importance of P. aeruginosa biofilms in CF, we investigated the effect of ibuprofen on GFP-expressing wild-type (WT) P. aeruginosa strain PAO1 (GFP-PAO1) in the biofilm growth mode *in vitro* (Fig. 5). Our studies were performed using a flow cell apparatus and confocal microscopy, which, coupled with the choice of GFP-PAO1 as a model biofilm-forming pathogen, allowed us to monitor these biofilms in real time and use voxel counts as a measure of biomass accumulation. Our results demonstrated that following an initial biomass accumulation during subexponential growth, a transition to exponential growth occurred for both treated and untreated biofilms; however, this transition was delayed in the presence of ibuprofen. This delay likely resulted from an increase in the surface-associated lag time when PAO1 planktonic cells made the transition to a biofilm growth mode, which was concomitant with the time required to change the expression levels of a large number of genes responsible for transitioning to the biofilm phenotype (67). Overall, our data match reasonably well those in reports in the literature, which documented the inhibition of biofilm formation by several strains of Streptococcus pneumoniae, E. coli, S. aureus, and Candida albicans following ibuprofen exposure (68–71). Mechanistically, the precise pathways by which ibuprofen exerts an antibiofilm effect are currently unknown, but several clues are available from the literature. For instance, diclofenac, ibuprofen, and salicylic acid have been shown to limit bacterial adhesion to abiotic surfaces (72–74). Exposure of E. coli to ibuprofen has been shown to impact bacterial adherence to epithelial cells by inhibition of fimbriae (70, 75) and through alterations to bacterial hydrophobicity and hemolysin production (70, 75), which impact biofilm formation. Therefore, there is a possibility that ibuprofen may have a similar effect on the ability of P. aeruginosa to produce biomolecules and undergo surface modification of the cell wall, thereby limiting biofilm formation (70–73). Additional evidence suggests that NSAIDs, including ibuprofen and salicylic acid, may be able to directly modulate quorum sensing, particularly through inhibition of the *las* system (76) regulating biofilm formation in P. aeruginosa, as well as also decrease the production of the virulence factors governed by these systems (77). Separately, since the manifestation of these biochemical processes requires ATP, the ability of ibuprofen to curtail ATP generation by acting as a protonophore may also be implicitly responsible for hindering the transition to a biofilm growth mode.

We examined the *in vivo* antimicrobial activity of ibuprofen in a mouse model of acute P. aeruginosa pneumonia. First, we demonstrated that the enteral delivery of 0.75 mg ibuprofen resulted in a peak serum ibuprofen concentration of 124.22 ± 15.40 μg/ml at 1 h after dosing. As described below, our dosing studies were guided by the work of Konstan et al. (16), in which they demonstrated a peak serum concentration of ibuprofen in rats at 1 h after either intraperitoneal or oral administration. Thereafter, mice were intranasally infected with an LD_50_ of P. aeruginosa (5 × 10^5^ CFU of strain PAO1) and treated with ibuprofen first at 2 h postinfection and subsequently at 8-h intervals. The effect of ibuprofen on the bacterial burden, as well as survival, was evaluated. At 36 h postinfection, ibuprofen-treated mice had significantly lower bacterial burdens in both the lungs and the spleen (Fig. 6A and B), as well as improved clinical scores (Fig. 6D). Subsequent survival experiments demonstrated significant improvements in not only the survival of the animals (Fig. 7A) but also weight loss and the clinical scores compared with those for the sham-treated animals (Fig. 7B and C), thus suggesting an overall improved health of ibuprofen-treated animals. In fact, only 1 out of the 13 mice in the ibuprofen group died of infection over a period of 3 days. In contrast, 6 deaths were recorded among the 14 sham-treated animals over the same time frame. As far as we are aware, this is the first report documenting a reduced bacterial burden as well as improved health parameters and survival in a P. aeruginosa pneumonia model following ibuprofen treatment.

In their seminal work, Konstan et al. observed improved weight gain and reduced inflammation, but no effect on bacterial burden, in a rat model of chronic pulmonary P. aeruginosa infection after treatment with ibuprofen (16). While we recognize the close interplay between bacterial infection and the subsequent inflammatory and immune responses and ibuprofen's ability to modulate both as important factors influencing the *in vivo* outcome in our studies, we suggest an additional yet unrecognized antimicrobial effect of ibuprofen *in vivo* as well, based on recent observations on the direct effect of ibuprofen on bacterial pathogens. We acknowledge, however, that our studies do not provide incontrovertible proof of this activity *in vivo*. Moreover, we acknowledge that the dissimilarity of our acute P. aeruginosa pneumonia model to the chronic biofilm infections in the cystic fibrosis lung is a limitation of this study. Additional studies are under way to address the challenging task of delineating the individual effect of ibuprofen on each of these systems *in vivo* to tweeze out the direct antimicrobial effects of ibuprofen.

Unfortunately, despite the advantages of ibuprofen therapy in CF patients, the utility has been limited due to concerns over safety, particularly gastrointestinal hemorrhage and nephrotoxicity, even though such occurrences are extremely rare (6). Additionally, the need to establish the dose in each patient on the basis of the results of pharmacokinetic studies has also been reported to be an impediment (78). Therefore, developing a novel aerosolizable formulation of ibuprofen would be highly advantageous, since such a formulation could significantly reduce the amount of drug necessary to achieve a clinically relevant outcome. Indeed, a few reports have demonstrated that the pulmonary delivery of NSAIDs reduces the dose needed to achieve anti-inflammatory efficacy by 3 to 6 orders of magnitude compared with the oral dose required (79, 80). Therefore, we first delivered by aerosol a water-soluble formulation of ibuprofen containing 1% (vol/vol) DMSO to healthy mice, which resulted in approximately 1% of the nebulized dose being delivered to each mouse; however, we encountered two main challenges. First, the low polarity of the solution resulted in poor aerosolization performance, thereby resulting in an excessive aerosolization time. Additionally, the residence time of the drug in the lung was found to be very low, as most of the delivered ibuprofen was found in the blood serum rather than in the BALF and lung tissue at 1 h postnebulization (Fig. 8). The rapid transport of small hydrophobic molecules from the lungs into the systemic circulation has been reported, owing to the huge surface area of the lungs, the highly dispersed nature of an aerosol, good epithelial permeability, and the small aqueous volume at the absorptive surface (81, 82). Therefore, to improve the residence time of ibuprofen in the lung, we developed nanoparticulate formulations of ibuprofen which can provide a depot delivery to the lung following nebulization. The nanoparticles were formulated using PLGA and PLGA-PEG, two commonly used biodegradable and biocompatible FDA-approved polymers with a wide range of applications in human therapy. Furthermore, the incorporation of PEG has been shown to increase the diffusion of nanoparticles through human mucoid surfaces (83), which, in the case of CF patients, might be particularly advantageous. As expected, all nanoparticles demonstrated spherical particles with a bimodal size distribution, minimal aggregation, and a mostly smooth surface morphology with some surface defects (Fig. 9), which are characteristic of nanoparticles formulated using an emulsion method. However, the nanoparticles formulated using NaIbu in the external phase demonstrated more surface defects, which could likely be attributed to the leaching of NaIbu from the particle surface during the washing steps. Separately, these particles also had the tightest size distribution, with the largest population being submicron-sized particles, suggesting that the presence of NaIbu in the emulsion modulates the particle diameter by influencing interfacial tension. The addition of NaIbu to the external phase during the formulation process also led to an exceedingly high ibuprofen load within the nanoparticles by reducing the oil-to-water-phase concentration gradient of the drug, thereby minimizing the diffusion of ibuprofen from within the nanoparticles. Lastly, through a preliminary study performed using strain PAO1, we have demonstrated a reduction in the CFU counts compared with those for the controls (bacteria treated with empty nanoparticles and medium only) following exposure to ibuprofen nanoparticles, thus validating the antimicrobial activity of these formulations (Fig. 10). We acknowledge that the reduction in the bacterial burden after exposure to ibuprofen nanoparticles was not as robust as that observed with nonformulated ibuprofen (∼0.6 log_10_ for Ibu/NaIbu-NPs versus ∼1.5 to 2.0 log_10_ for 100 μg/ml unformulated ibuprofen); however, this preliminary study was performed primarily to demonstrate a proof of concept. For the current nanoparticle formulations, the dosing was calculated on the basis of the ibuprofen load alone without factoring in the release rates. Further, these nanoparticles do not possess optimal ibuprofen loading characteristics and release rates. We are currently optimizing the nanoparticle formulation parameters as well as processes to enhance ibuprofen loading, reduce the particle size distribution, modify the surface characteristics, and modulate the release kinetics as well as pharmacokinetics to obtain the best nanoparticle system for aerosolization. A thorough characterization of these formulations is concurrently under way and will allow us to investigate the antimicrobial activity of the nanoparticle formulations systematically.

In summary, we have demonstrated the direct antimicrobial activity of ibuprofen on strains of two important CF-associated Gram-negative bacterial pathogens *in vitro* under a variety of conditions mimicking the environment in the CF lung. We also confirmed the conservation of this activity *in vivo* in a mouse model of acute P. aeruginosa pneumonia and demonstrated a reduced bacterial burden and an improvement in the overall health of the ibuprofen-treated animals, which led to a superior survival outcome. Based on the conclusions derived from reports previously published in the literature and our data, we suggest that the beneficial effects of ibuprofen observed in clinical trials in patients with CF are due to a combination of its antimicrobial and anti-inflammatory effects and not solely its anti-inflammatory effects, as previously believed. Furthermore, to increase the utility of ibuprofen therapy in CF, we have developed an aerosolizable nanoparticle formulation of ibuprofen capable of depot delivery to the lung. This approach may be able to provide the target therapeutic dose locally, thereby preserving the beneficial clinical effects while potentially mitigating the toxicity and pharmacokinetic concerns associated with high systemic concentrations resulting from traditional oral therapy. We believe that the robust anti-inflammatory activity of ibuprofen, coupled with its mild to moderate antimicrobial activity against multidrug-resistant pathogens, makes it an extremely attractive candidate as an adjunct therapeutic and hope that our findings will provide an impetus for the adoption of this therapeutic as standard treatment by more CF centers globally.

## MATERIALS AND METHODS

### Bacterial strains.

Both CF and non-CF strains of Pseudomonas aeruginosa and Burkholderia spp. were utilized for our studies and comprised laboratory as well as clinical isolates. P. aeruginosa laboratory strain PAO1 was generously donated by Gerald Pier (Harvard University, Boston, MA), whereas the CF mucoid clinical isolate P. aeruginosa M57-15 was generously donated by Thomas Ferkol (Washington University, St. Louis, MO) (84). The remaining two P. aeruginosa CF clinical isolates (strain T63547 and H25815, both of which were from sputum) were obtained from the American Type Culture Collection. Burkholderia cenocepacia K56-2 is a CF clinical isolate which has been previously characterized (85) and was obtained from BEI Resources (catalog number NR-20535; Manassas, VA). The remaining isolates of Burkholderia spp., including Burkholderia cenocepacia (CF clinical isolate HI2477 from sputum), Burkholderia cepacia (clinical isolate 1753), and Burkholderia multivorans (chronic granulomatous disease [CGD] clinical isolate SH-2 from a biopsy specimen) were obtained from the culture collection of the Microbiology Laboratory at the National Institutes of Health (NIH) Clinical Center (Bethesda, MD) and were identified as previously described using 16S rRNA gene- and *recA*-targeted PCR assays (86, 87).

### *In vitro* antimicrobial activity of ibuprofen.

Bacteria were streaked from frozen glycerol stocks onto tryptic soy agar (TSA) or tryptic soy agar with 5% sheep blood plates (Remel) and incubated overnight at 37°C with 5% carbon dioxide (CO_2_) until individual colonies formed. A single colony was inoculated in 10 ml MH broth and grown at 37°C in a shaking incubator overnight to stationary phase. The cultures were harvested and washed three times with 150 mM NaCl solution and diluted to a density of 5 × 10^5^ CFU/ml in MH broth. A 10-mg/ml stock solution of ibuprofen (in DMSO) was used to prepare working solutions of ibuprofen at 100, 150, and 200 μg/ml in MH broth containing DMSO (5%, vol/vol). For studies in 96-well plates, 100 μl of bacterial culture was added to ibuprofen working solutions (100 μl), resulting in final ibuprofen concentrations of 50, 75, and 100 μg/ml in a total volume of 200 μl. Uninoculated MH broth and MH broth with DMSO (5%, vol/vol) without ibuprofen were used as controls for the experiment. The final DMSO concentration in all samples was 2.5% (vol/vol). After inoculation, the prepared culture solutions were incubated at 37°C with CO_2_ for 12 h. Subsequently, the number of viable cells was analyzed by plating each culture solution (0.05 ml) after dilution (1:10) with 150 mM NaCl solution, incubating at 37°C with CO_2_, and counting the number of CFU. Antimicrobial activity determination studies were performed in duplicate wells in at least three replicate experiments, and the data were pooled for analysis.

### Bacterial spiking with ibuprofen.

Strains of Pseudomonas aeruginosa (PAO1 and M57-15), Burkholderia cenocepacia (K56-2), and Burkholderia multivorans were grown and adjusted to a density of 5 × 10^5^ CFU/ml in MH broth as described above. Ibuprofen and control samples were also prepared as described above and incubated with 100 μl of bacterial culture, resulting in final test concentrations of 50, 75, and 100 μg/ml in a total volume of 200 μl. After inoculation, the prepared culture solutions were incubated at 37°C with CO_2_ for 12 h. At 12 h, the samples were spiked with 10 μl of either MH broth with DMSO (5%, vol/vol) or ibuprofen in DMSO to achieve the initial concentration and incubated for an additional 6 h (18-h time point). The new volume of 210 μl per well was subsequently used to quantify the number of CFU per milliliter. Growth was performed in duplicate wells in at least four replicate experiments. The number of viable cells was determined by plating and counting the number of CFU as described above.

### Activity of ibuprofen in artificial CF sputum.

Burkholderia cenocepacia strain K56-2 and Pseudomonas aeruginosa strain PAO1 were streaked, cultured, and harvested according to the methods described above. The bacteria were diluted to a density of 1 × 10^6^ CFU/ml in MH broth or artificial sputum medium (ASM), which was prepared by use of the recipe by Sriramulu et al. (48). Two-hundred-microliter cultures containing bacteria alone in the presence or absence of DMSO (positive controls) or 100 μg/ml ibuprofen (in medium containing DMSO for ibuprofen dissolution) were grown in duplicate in 96-well plates at 37°C. Blank sample wells with uninoculated medium served as negative controls. DMSO was maintained at a final concentration of 2.5% (vol/vol) in all DMSO-containing samples. The absorbance (optical density at 600 nm [OD_600_]) was monitored at 1-h intervals over a period of 12 h and 24 h, after which the samples were plated to determine bacterial growth (the number of CFU).

### *In vitro* biofilm studies.

Wild-type (WT) Pseudomonas aeruginosa PAO1 expressing green fluorescent protein (GFP) on plasmid pMRP9-1 was used for confocal experiments (58). P. aeruginosa was streaked from a frozen stock onto LB agar (5 g/liter yeast extract, 5 g/liter casein peptone, 10 g/liter sodium chloride, 15 g/liter agar) plates and incubated overnight at 37°C. Three or four colonies were then inoculated into a culture tube containing 4 ml fastidious anaerobe broth (FAB) medium (88) with 3% (vol/vol) tryptic soy broth (TSB) and 30 mM d-glucose as the carbon source. Liquid cultures were grown at 37°C on an orbital shaker (Labnet Orbit 1000) operating at 200 to 250 rpm. The LB agar and liquid culture media were supplemented with 150 μg/ml carbenicillin for plasmid maintenance; however, for use in flow cells, the FAB medium was adjusted to contain 3% (vol/vol) TSB and 0.3 mM d-glucose without carbenicillin. For the ibuprofen exposure experiments, the FAB medium to be used in the flow cell was supplemented with ibuprofen at a final concentration of 100 μg/ml. For the control experiments, FAB medium without ibuprofen but with the appropriate quantity of DMSO was used. To perform experiments in flow cells, liquid cultures were grown in culture tubes to an OD_600_ of 0.3 (corresponding to mid-exponential growth phase), as measured by a Thermo Spectronic Genesys 20 spectrophotometer. The culture was then volumetrically diluted to an OD_600_ of approximately 0.0015 for inoculation into the flow cell chambers. Experiments were performed in a standard three-channel flow cell system (89). The flow cell systems were sterilized by autoclaving prior to mounting the flow cell and bubble trap on the microscope stage. Subsequently, the flow cells were filled with sterile FAB medium and ∼1 ml of inoculum was injected via a needle and syringe into each chamber of the flow cell. The cells were allowed to remain for 1 h under static conditions to facilitate initial attachment, after which FAB medium with or without ibuprofen was perfused through the flow cell at a rate of 65 μl/min using a Watson-Marlow peristaltic pump, and image capture was simultaneously begun.

Confocal laser scanning microscopy was performed on an Olympus FV1000 motorized inverted IX81 microscope suite with a 40× air objective. The stage was enclosed with an incubation chamber to allow temperature control of the sample and was maintained at 30°C. Image capture was controlled by FV10-ASW (version 3.1) software. Confocal z-stacks were taken during 2-min scans in the GFP channel using a 488-nm excitation laser. Images were analyzed using the Fiji distribution of ImageJ software (90), and voxel counting was performed with custom-written codes in MATLAB, as previously described in articles by Hutchison et al. (91) and Kragh et al. (92). Here, the voxel counts serve as a measure of biomass accumulation in the biofilm, since bacteria not attached to the surface are transported by the flowing medium and are specifically excluded by the custom-written MATLAB code (91, 92). Exponential parts of voxel count curves were analyzed by least-squares regression (Microsoft Excel software), and the equation *B* = α*e^t^*^/τ^ (where *B* is the level of biomass accumulation [measured in voxels], α is the initial biomass at the onset of exponential growth [typically, 14,000 to 22,000 voxels], *t* is the amount of time that has elapsed from the onset of exponential growth, and τ is a characteristic growth time scale) was used to calculate the doubling time, which is τ ln(2). Further refinement of this calculation was performed by fitting an exponential function, *d*^2^(voxels)/*dt*^2^ ≡ *d*^2^*B*/*dt*^2^ = *B̈*_0_*e^t^*^/τ^, to the second derivative with respect to the time of voxel counting for the exponential regime of these data for each biofilm. The second derivative measures the rate at which the biofilm growth rate is increasing and gives a more sensitive indication of the exponential growth regime than the voxel counts or their first derivative. The fit function was extrapolated backward in time to time zero to determine ¨*B*_0_, which is equivalent to α/τ^2^. The fit functions were then compared for pairs of control and ibuprofen-treated biofilms that were grown in parallel experiments on the same day.

### Determination of intracellular ATP concentration in bacteria.

P. aeruginosa strain PAO1 was grown as previously described. A planktonic suspension of PAO1 in MH broth containing DMSO (10^8^ CFU/ml) was incubated in a shaking incubator (37°C, 200 rpm) in the absence (control) or presence of ibuprofen (100 μg/ml) for 30 min. After incubation, part of the suspension was plated on agar for determination of the number of CFU, while the remainder was immediately frozen at −80°C to lyse the bacteria and then thawed at room temperature. Subsequently, ATP was extracted by addition of a solution of 0.5% Triton X-100, 25 mM HEPES, and 2 mM EDTA (pH 7.8) in a 1:1 ratio. The ATP content was measured using a spectrophotometer and the Molecular Probes ATP determination kit (Invitrogen), which utilizes firefly luciferase and d-luciferin to generate a bioluminescent signal, whose intensity is proportional to the ATP concentration.

### Investigation of ibuprofen treatment in a murine acute P. aeruginosa pneumonia model.

Male C57BL/6J mice (The Jackson Laboratory, Bar Harbor, ME) at 6 to 8 weeks of age were used for all studies, and the animals were housed in a barrier facility under pathogen-free conditions until inoculation with bacteria. The studies were approved by the University of Texas Southwestern Medical Center Institutional Animal Care and Use Committee (IACUC). Ibuprofen (catalog number I0415; Tokyo Chemical Industry Co. Ltd., Tokyo, Japan) was suspended in a 1:1 (vol/vol) solution of strawberry-flavored syrup (Smucker's) and sterile distilled, deionized water (DH_2_O) at a concentration of 7.5 mg/ml. Similarly, a sham treatment solution was prepared by mixing a 1:1 (vol/vol) solution of strawberry-flavored syrup and DH_2_O. Both treatment solutions were vortexed for 3 min and sonicated for 1 min at 70% amplitude (Sonics Vibra-cell VCX130; Sonics & Materials, Inc., Newtown, CT).

Uninfected C57BL/6J mice were fed via a syringe with one dose of 100 μl of either the ibuprofen suspension or the sham treatment solution (*n* = 4). At 1 h posttreatment, the mice were anesthetized, dissected, and euthanized via cardiac puncture. The rationale for sampling blood serum at 1 h after ibuprofen administration was derived from the data previously published by Konstan et al., who demonstrated that ibuprofen reaches peak serum concentrations at 1 h and reaches baseline values at approximately 6 h following intraperitoneal and oral administration in rats (16). Similar results have also been observed in several human studies summarized by Davies (58). Blood from each mouse was collected in separate serum separator tubes (Greiner Bio-One, Monroe, NC) and centrifuged at 1,000 × *g* for 10 min to collect the serum. The ibuprofen concentrations in the serum were determined using an enzyme-linked immunosorbent assay (ELISA) kit (Neogen) according to the manufacturer's instructions.

Acute lung infection studies in mice were performed using a standard laboratory strain of P. aeruginosa, strain PAO1. The bacteria were grown on TSA plates as described above, after which a single colony was suspended in Luria broth (LB; 10 ml) and grown in a shaking incubator (37°C, 200 rpm) to an OD_650_ of 0.4, corresponding to a bacterial load of ∼2 × 10^8^ to 3 × 10^8^ CFU/ml, as determined by serial dilution and plating. The first infection model experiment evaluated the ability of ibuprofen to reduce the bacterial burden in mice inoculated with PAO1. Following anesthesia, mice were intranasally inoculated with 75 μl of PAO1 in LB broth at an LD_50_ of ∼5 × 10^5^ CFU per mouse. Subsequently, the mice were weighed and randomly assigned to one of the two treatment groups. At 2 h postinoculation and every 8 h thereafter, the mice were fed via a syringe 100 μl of either the ibuprofen suspension (0.75 mg ibuprofen) or the sham treatment solution. At 36 h postinoculation, the mice were euthanized (by sedation with tribromoethanol [Avertin] and cervical dislocation) and their lungs and spleens were harvested, homogenized, and plated onto TSA plates to determine CFU counts. The mice were also weighed and assigned clinical scores at the start of the study, at 12 h, and at 36 h (termination of the study). The clinical scores are a semiquantitative metric that rates the signs of infection from asymptomatic (score, 0) to moribund (score, 6) on the basis of three parameters: activity, fur, and posture (93). The experiment was performed in triplicate, and data from each experiment were pooled. A second experiment was performed to evaluate the effect of ibuprofen on the survival of PAO1-infected mice using the same protocol used for the previous experiment. However, the mice were monitored for a total of 72 h after inoculation, and the time of death for each mouse was recorded immediately. Treatments were also continued at 8-h intervals up to 64 h postinoculation. The mice were weighed and assigned a clinical score at the start of the experiment and subsequently at 24-h intervals. The survival experiment was performed in duplicate, and the data from each experiment were pooled.

### Aerosol delivery of ibuprofen to mice and determination of preliminary biodistribution.

An aerosolizable formulation of ibuprofen was prepared by dissolving either 25 or 50 mg ibuprofen in a solution containing 1% (vol/vol) DMSO and phosphate (PO_4_) buffer. Healthy mice were randomly distributed into three groups (3 mice per group) and provided with one of the following aerosolized treatment: 25 mg ibuprofen, 50 mg ibuprofen, or vehicle. Aerosolization was achieved using an Aeroneb lab apparatus (Aerogen Inc., Galway, Ireland) connected to a multidosing animal chamber, which is a square Plexiglas box with inner dimensions of 8 in. in length, 8 in. in width, and 6 in. in height, with the nebulizer being mounted in the center of the lid. The Aeroneb nebulizer is based on a micropump technology that produces droplets (2.5 to 4 μm) in a low-velocity aerosol (94). The mice were individually placed into CH-247 restraint tubes (CH Technologies, Westwood, NJ) and then placed inside the multidosing chamber to provide aerosolized treatment. Immediately following the nebulization treatment, the mice were anesthetized by an intraperitoneal injection of tribromoethanol, and complete sedation was verified by toe pinch. The mice were then dissected and euthanized by cardiac puncture, and blood was collected in serum separator tubes (BD, Franklin Lakes, NJ) and centrifuged at 1,000 × *g* for 10 min to collect the serum. Bronchoalveolar lavage fluid (BALF) was collected by the injection and aspiration of 3 ml of sterile PO_4_ buffer into the lungs via the trachea using a catheter and syringe. Next, the whole lung tissue was harvested and homogenized in 1 ml of sterile PO_4_ buffer. The harvested serum, BALF, and lung tissue were analyzed for urea content (BioVision, Inc., Milpitas, CA), which allows the concentrations of ibuprofen (or other drugs) to be normalized to a true volumetric concentration, since urea concentrations should be constant throughout the body. Finally, the ibuprofen concentrations against a standard of ibuprofen dissolved in the 99% PO_4_ buffer–1% DMSO solution used for nebulization were determined using an ELISA kit (Neogen).

### Formulation of ibuprofen nanoparticles and determination of preliminary *in vitro* activity.

Ibuprofen nanoparticles (NPs) were formulated using the oil-in-water (o/w) emulsion technique from a mixture of two biodegradable polymers: poly(lactide-co-glycolide) (PLGA) and a copolymer of PLGA and polyethylene glycol (PEG) (PLGA-PEG). The polymers were dissolved in 3 ml chloroform (100 mg/ml) along with ibuprofen (10 mg/ml), and the organic solution was emulsified with 100 ml polyvinylpyrrolidone (PVP) solution (10% [wt/vol] in water) loaded with or without the sodium salt of ibuprofen for 3 min with high-speed mechanical stirring (∼2,400 rpm), after which the emulsion was gently stirred (∼300 rpm) at room temperature for 5 h to remove the organic solvent. The resulting nanoparticle suspension was centrifuged, washed with sterile distilled, deionized water (DH_2_O), shell frozen, and lyophilized to obtain the NPs as a free-flowing powder. Subsequently, the NPs were characterized for surface morphology and size distribution using standard scanning electron microscopy (SEM) techniques and an FEI XL30 environmental scanning electron microscope at an accelerating voltage of 15 kV. The size distribution of the NPs was determined by analyzing 100 random nanoparticles on SEM images using an appropriate scaling factor (ImageJ software; NIH, Bethesda, MD). The ibuprofen load within the nanoparticles was determined by dissolving the NPs in chloroform, diluting 50 times in sterile DH_2_O, and measuring the ibuprofen content with an ELISA kit (Neogen).

The antimicrobial activity of ibuprofen nanoparticles was determined using a study of the endpoint number of CFU. Planktonic suspensions of strain PAO1 at 5 × 10^5^ CFU/ml in MH broth were incubated with ibuprofen nanoparticles, empty nanoparticles, or a control solution (MH broth only). The concentration of the nanoparticles used for this study was determined on the basis of the ibuprofen load (theoretical ibuprofen concentration, 500 μg/ml), and an equivalent mass of empty nanoparticles was used. The suspensions were placed on a shaking incubator for 6 h (37°C, 200 rpm), after which the CFU counts were determined by plating. The experiments were performed in duplicate, with three technical replicates being used for each experiment (total, 6 replicate samples per treatment).

### Statistics.

Analyses were performed using Prism (version 6) software (GraphPad Software, Inc., San Diego, CA). Data are presented as the mean ± standard error of the mean. The endpoint differences for growth curves (OD_600_) were analyzed using a two-tailed paired *t* test. The CFU counts obtained from most *in vitro* studies, the differences in the ATP concentration/number of CFU of bacteria, as well as the differences in the bacterial burden between the lungs and spleens, weight differentials, and differences in the clinical scores between ibuprofen- and sham-treated mice, were analyzed using a two-tailed, nonparametric Mann-Whitney test. The serum, lung, and BALF concentrations of ibuprofen following the aerosol delivery of various doses of ibuprofen were compared using a two-way analysis of variance (ANOVA) with Tukey's multiple-comparison *post hoc* test. In the case of the CFU counts obtained from the ibuprofen NP experiments, the data were analyzed using ordinary one-way ANOVA with Tukey's multiple-comparison *post hoc* test. The *in vivo* survival curves in the infection model were compared using a log-rank Mantel-Cox test. Data were considered to exhibit statistically significant differences when *P* was <0.05.

## Supplementary Material

Supplemental material

## References

[1] Chmiel JF, Berger M, Konstan MW 2002 The role of inflammation in the pathophysiology of CF lung disease. Clin Rev Allergy Immunol 23:5–27. doi:10.1385/CRIAI:23:1:005. 12162106

[2] Davis PB, Drumm M, Konstan MW 1996 Cystic fibrosis: state of the art. Am J Respir Crit Care Med 154:1229–1256. doi:10.1164/ajrccm.154.5.8912731. 8912731

[3] Dakin CJ, Numa AH, Wang H, Morton JR, Vertzyas CC, Henry RL 2002 Inflammation, infection, and pulmonary function in infants and young children with cystic fibrosis. Am J Respir Crit Care Med 165:904–910. doi:10.1164/ajrccm.165.7.2010139. 11934712

[4] Chmiel JF, Konstan MW 2007 Inflammation and anti-inflammatory therapies for cystic fibrosis. Clin Chest Med 28:331–346. doi:10.1016/j.ccm.2007.02.002. 17467552

[5] Dauletbaev N, Lam J, Eklove D, Iskandar M, Lands LC 2010 Ibuprofen modulates NF-κB activity but not IL-8 production in cystic fibrosis respiratory epithelial cells. Respiration 79:234–242. doi:10.1159/000255342. 19887769

[6] Nichols DP, Konstan MW, Chmiel JF 2008 Anti-inflammatory therapies for cystic fibrosis-related lung disease. Clin Rev Allergy Immunol 35:135–153. doi:10.1007/s12016-008-8081-2. 18546078

[7] Konstan MW 2008 Ibuprofen therapy for cystic fibrosis lung disease: revisited. Curr Opin Pulm Med 14:567–573. doi:10.1097/MCP.0b013e32831311e8. 18812834

[8] Cheng K, Ashby D, Smyth R 2011 Oral steroids for long-term use in cystic fibrosis. Cochrane Database Syst Rev 10:CD000407. doi:10.1002/14651858.CD000407.pub2. 21975730

[9] Lai HC, FitzSimmons SC, Allen DB, Kosorok MR, Rosenstein BJ, Campbell PW, Farrell PM 2000 Risk of persistent growth impairment after alternate-day prednisone treatment in children with cystic fibrosis. N Engl J Med 342:851–859. doi:10.1056/NEJM200003233421204. 10727589

[10] Konstan MW, Byard PJ, Hoppel CL, Davis PB 1995 Effect of high-dose ibuprofen in patients with cystic fibrosis. N Engl J Med 332:848–854. doi:10.1056/NEJM199503303321303. 7503838

[11] Saiman L, Marshall BC, Mayer-Hamblett N, Burns JL, Quittner AL, Cibene DA, Coquillette S, Fieberg AY, Accurso FJ, Campbell PW III 2003 Azithromycin in patients with cystic fibrosis chronically infected with Pseudomonas aeruginosa: a randomized controlled trial. JAMA 290:1749–1756. doi:10.1001/jama.290.13.1749. 14519709

[12] Lands LC, Milner R, Cantin AM, Manson D, Corey M 2007 High-dose ibuprofen in cystic fibrosis: Canadian safety and effectiveness trial. J Pediatr 151:249–254. doi:10.1016/j.jpeds.2007.04.009. 17719932

[13] Lands LC, Stanojevic S 2013 Oral non-steroidal anti-inflammatory drug therapy for lung disease in cystic fibrosis. Cochrane Database Syst Rev 6:CD001505. doi:10.1002/14651858.CD001505.pub3. 23765216

[14] Sordelli DO, Cerquetti MC, el-Tawil G, Ramwell PW, Hooke AM, Bellanti JA 1985 Ibuprofen modifies the inflammatory response of the murine lung to Pseudomonas aeruginosa. Eur J Respir Dis 67:118–127. 3863757

[15] Rinaldo JE, Pennock B 1986 Effects of ibuprofen on endotoxin-induced alveolitis: biphasic dose response and dissociation between inflammation and hypoxemia. Am J Med Sci 291:29–38. doi:10.1097/00000441-198601000-00007. 2934983

[16] Konstan MW, Vargo KM, Davis PB 1990 Ibuprofen attenuates the inflammatory response to Pseudomonas aeruginosa in a rat model of chronic pulmonary infection. Implications for antiinflammatory therapy in cystic fibrosis. Am Rev Respir Dis 141:186–192. 2153353 10.1164/ajrccm/141.1.186

[17] Konstan MW, Schluchter MD, Xue W, Davis PB 2007 Clinical use of ibuprofen is associated with slower FEV1 decline in children with cystic fibrosis. Am J Respir Crit Care Med 176:1084–1089. doi:10.1164/rccm.200702-181OC. 17872492 PMC2176097

[18] Brown KA, Collins AJ 1977 Action of nonsteroidal, anti-inflammatory drugs on human and rat peripheral leucocyte migration in vitro. Ann Rheum Dis 36:239–243. doi:10.1136/ard.36.3.239. 301730 PMC1006672

[19] Kaplan HB, Edelson HS, Korchak HM, Given WP, Abramson S, Weissmann G 1984 Effects of non-steroidal anti-inflammatory agents on human neutrophil functions in vitro and in vivo. Biochem Pharmacol 33:371–378. doi:10.1016/0006-2952(84)90228-4. 6422946

[20] Maderazo EG, Breaux SP, Woronick CL 1984 Inhibition of human polymorphonuclear leukocyte cell responses by ibuprofen. J Pharm Sci 73:1403–1406. doi:10.1002/jps.2600731020. 6502489

[21] Shimanuki T, Nakamura RM, Dizerega GS 1985 Modulation of leukotaxis by ibuprofen. A quantitative determination in vivo. Inflammation 9:285–295. 3930400 10.1007/BF00916277

[22] Venezio FR, DiVincenzo C, Pearlman F, Phair JP 1985 Effects of the newer nonsteroidal anti-inflammatory agents, ibuprofen, fenoprofen, and sulindac, on neutrophil adherence. J Infect Dis 152:690–694. doi:10.1093/infdis/152.4.690. 4045229

[23] Sanyal AK, Roy D, Chowdhury B, Banerjee AB 1993 Ibuprofen, a unique anti-inflammatory compound with antifungal activity against dermatophytes. Lett Appl Microbiol 17:109–111. doi:10.1111/j.1472-765X.1993.tb01436.x.

[24] Elvers KT, Wright SJ 1995 Antibacterial activity of the anti-inflammatory compound ibuprofen. Lett Appl Microbiol 20:82–84. doi:10.1111/j.1472-765X.1995.tb01291.x. 7765904

[25] Shirin H, Moss SF, Kancherla S, Kancherla K, Holt PR, Weinstein IB, Sordillo EM 2006 Non-steroidal anti-inflammatory drugs have bacteriostatic and bactericidal activity against Helicobacter pylori. J Gastroenterol Hepatol 21:1388–1393. 16911681 10.1111/j.1440-1746.2006.04194.x

[26] Hersh EV, Hammond BF, Fleury AAP 1991 Antimicrobial activity of flurbiprofen and ibuprofen *in vitro* against six common periodontal pathogens. J Clin Dent 3:1–5. 1812907

[27] Obad J, Suskovic J, Kos B 2015 Antimicrobial activity of ibuprofen: new perspectives on an “old” non-antibiotic drug. Eur J Pharm Sci 71:93–98. doi:10.1016/j.ejps.2015.02.011. 25708941

[28] Guzman JD, Evangelopoulos D, Gupta A, Birchall K, Mwaigwisya S, Saxty B, McHugh TD, Gibbons S, Malkinson J, Bhakta S 2013 Antitubercular specific activity of ibuprofen and the other 2-arylpropanoic acids using the HT-SPOTi whole-cell phenotypic assay. BMJ Open 3:e002672. doi:10.1136/bmjopen-2013-002672. PMC3693423 23794563

[29] Chan EWL, Yee ZY, Raja I, Yap JKY 2017 Synergistic effect of non-steroidal anti-inflammatory drugs (NSAIDs) on antibacterial activity of cefuroxime and chloramphenicol against methicillin-resistant Staphylococcus aureus. J Glob Antimicrob Resist 10:70–74. doi:10.1016/j.jgar.2017.03.012. 28673701

[30] Pina-Vaz C, Sansonetty F, Rodrigues AG, Martinez-De-Oliveira J, Fonseca AF, Mardh PA 2000 Antifungal activity of ibuprofen alone and in combination with fluconazole against Candida species. J Med Microbiol 49:831–840. doi:10.1099/0022-1317-49-9-831. 10966233

[31] Scott EM, Tariq VN, McCrory RM 1995 Demonstration of synergy with fluconazole and either ibuprofen, sodium salicylate, or propylparaben against Candida albicans in vitro. Antimicrob Agents Chemother 39:2610–2614. doi:10.1128/AAC.39.12.2610. 8592988 PMC162998

[32] Tariq VN, Scott EM, McCain NE 1995 Use of decimal assay for additivity to demonstrate synergy in pair combinations of econazole, nikkomycin Z, and ibuprofen against Candida albicans in vitro. Antimicrob Agents Chemother 39:2615–2619. doi:10.1128/AAC.39.12.2615. 8592989 PMC162999

[33] Byrne ST, Denkin SM, Zhang Y 2007 Aspirin and ibuprofen enhance pyrazinamide treatment of murine tuberculosis. J Antimicrob Chemother 59:313–316. doi:10.1093/jac/dkl486. 17185297

[34] del Prado G, Martinez-Marin C, Huelves L, Gracia M, Rodriguez-Cerrato V, Fernandez-Roblas R, Ponte C, Cenjor C, Soriano F 2006 Impact of ibuprofen therapy in the outcome of experimental pneumococcal acute otitis media treated with amoxicillin or erythromycin. Pediatr Res 60:555–559. doi:10.1203/01.PDR.0000242258.52590.b5. 16966357

[35] Singh PK, Schaefer AL, Parsek MR, Moninger TO, Welsh MJ, Greenberg EP 2000 Quorum-sensing signals indicate that cystic fibrosis lungs are infected with bacterial biofilms. Nature 407:762–764. doi:10.1038/35037627. 11048725

[36] Drenkard E 2003 Antimicrobial resistance of Pseudomonas aeruginosa biofilms. Microbes Infect 5:1213–1219. doi:10.1016/j.micinf.2003.08.009. 14623017

[37] Livermore DM 2002 Multiple mechanisms of antimicrobial resistance in Pseudomonas aeruginosa: our worst nightmare? Clin Infect Dis 34:634–640. doi:10.1086/338782. 11823954

[38] Oliver A, Canton R, Campo P, Baquero F, Blazquez J 2000 High frequency of hypermutable Pseudomonas aeruginosa in cystic fibrosis lung infection. Science 288:1251–1254. doi:10.1126/science.288.5469.1251. 10818002

[39] Henwood CJ, Livermore DM, James D, Warner M, Pseudomonas Study Group. 2001 Antimicrobial susceptibility of Pseudomonas aeruginosa: results of a UK survey and evaluation of the British Society for Antimicrobial Chemotherapy disc susceptibility test. J Antimicrob Chemother 47:789–799. doi:10.1093/jac/47.6.789. 11389111

[40] Mahenthiralingam E, Baldwin A, Vandamme P 2002 Burkholderia cepacia complex infection in patients with cystic fibrosis. J Med Microbiol 51:533–538. doi:10.1099/0022-1317-51-7-533. 12132768

[41] Mahmud T, Rafi SS, Scott DL, Wrigglesworth JM, Bjarnason I 1996 Nonsteroidal antiinflammatory drugs and uncoupling of mitochondrial oxidative phosphorylation. Arthritis Rheum 39:1998–2003. doi:10.1002/art.1780391208. 8961904

[42] Krause MM, Brand MD, Krauss S, Meisel C, Vergin H, Burmester GR, Buttgereit F 2003 Nonsteroidal antiinflammatory drugs and a selective cyclooxygenase 2 inhibitor uncouple mitochondria in intact cells. Arthritis Rheum 48:1438–1444. doi:10.1002/art.10969. 12746918

[43] Terada H 1990 Uncouplers of oxidative phosphorylation. Environ Health Perspect 87:213–218. doi:10.1289/ehp.9087213. 2176586 PMC1567840

[44] Yin Z, Wang Y, Whittell LR, Jergic S, Liu M, Harry E, Dixon NE, Kelso MJ, Beck JL, Oakley AJ 2014 DNA replication is the target for the antibacterial effects of nonsteroidal anti-inflammatory drugs. Chem Biol 21:481–487. doi:10.1016/j.chembiol.2014.02.009. 24631121

[45] Denkin S, Byrne S, Jie C, Zhang Y 2005 Gene expression profiling analysis of Mycobacterium tuberculosis genes in response to salicylate. Arch Microbiol 184:152–157. doi:10.1007/s00203-005-0037-9. 16175359

[46] Kahlous NA, Bawarish MAM, Sarhan MA, Kupper M, Hasaba A, Rajab M 2017 Using chemoinformatics, bioinformatics, and bioassay to predict and explain the antibacterial activity of nonantibiotic Food and Drug Administration drugs. Assay Drug Dev Technol 15:89–105. doi:10.1089/adt.2016.771. 28346800

[47] Legg TJ, Laurent AL, Leyva R, Kellstein D 2014 Ibuprofen sodium is absorbed faster than standard ibuprofen tablets: results of two open-label, randomized, crossover pharmacokinetic studies. Drugs R D 14:283–290. doi:10.1007/s40268-014-0070-8. 25395311 PMC4269818

[48] Sriramulu DD, Lunsdorf H, Lam JS, Romling U 2005 Microcolony formation: a novel biofilm model of Pseudomonas aeruginosa for the cystic fibrosis lung. J Med Microbiol 54:667–676. doi:10.1099/jmm.0.45969-0. 15947432

[49] Clamens T, Rosay T, Crepin A, Grandjean T, Kentache T, Hardouin J, Bortolotti P, Neidig A, Mooij M, Hillion M, Vieillard J, Cosette P, Overhage J, O'Gara F, Bouffartigues E, Dufour A, Chevalier S, Guery B, Cornelis P, Feuilloley MG, Lesouhaitier O 2017 The aliphatic amidase AmiE is involved in regulation of Pseudomonas aeruginosa virulence. Sci Rep 7:41178. doi:10.1038/srep41178. 28117457 PMC5259723

[50] Rakhimova E, Munder A, Wiehlmann L, Bredenbruch F, Tummler B 2008 Fitness of isogenic colony morphology variants of Pseudomonas aeruginosa in murine airway infection. PLoS One 3:e1685. doi:10.1371/journal.pone.0001685. 18301762 PMC2246019

[51] Mann EE, Magin CM, Mettetal MR, May RM, Henry MM, DeLoid H, Prater J, Sullivan L, Thomas JG, Twite MD, Parker AE, Brennan AB, Reddy ST 2016 Micropatterned endotracheal tubes reduce secretion-related lumen occlusion. Ann Biomed Eng 44:3645–3654. doi:10.1007/s10439-016-1698-z. 27535564 PMC11949081

[52] Pompilio A, Crocetta V, Verginelli F, Di Bonaventura G 2016 In vitro activity of levofloxacin against planktonic and biofilm Stenotrophomonas maltophilia lifestyles under conditions relevant to pulmonary infection in cystic fibrosis, and relationship with SmeDEF multidrug efflux pump expression. FEMS Microbiol Lett 363:fnw145. doi:10.1093/femsle/fnw145. 27242375

[53] Housby JN, Cahill CM, Chu B, Prevelige R, Bickford K, Stevenson MA, Calderwood SK 1999 Non-steroidal anti-inflammatory drugs inhibit the expression of cytokines and induce HSP70 in human monocytes. Cytokine 11:347–358. doi:10.1006/cyto.1998.0437. 10328874

[54] Konstan MW, Krenicky JE, Finney MR, Kirchner HL, Hilliard KA, Hilliard JB, Davis PB, Hoppel CL 2003 Effect of ibuprofen on neutrophil migration in vivo in cystic fibrosis and healthy subjects. J Pharmacol Exp Ther 306:1086–1091. doi:10.1124/jpet.103.052449. 12807998

[55] Scheuren N, Bang H, Munster T, Brune K, Pahl A 1998 Modulation of transcription factor NF-kappaB by enantiomers of the nonsteroidal drug ibuprofen. Br J Pharmacol 123:645–652. doi:10.1038/sj.bjp.0701652. 9517383 PMC1565210

[56] Tegeder I, Pfeilschifter J, Geisslinger G 2001 Cyclooxygenase-independent actions of cyclooxygenase inhibitors. FASEB J 15:2057–2072. doi:10.1096/fj.01-0390rev. 11641233

[57] Zimmermann P, Curtis N 2017 Antimicrobial effects of antipyretics. Antimicrob Agents Chemother 61:e02268-16. doi:10.1128/AAC.02268-16. 28137805 PMC5365702

[58] Davies NM 1998 Clinical pharmacokinetics of ibuprofen. The first 30 years. Clin Pharmacokinet 34:101–154. doi:10.2165/00003088-199834020-00002. 9515184

[59] Langenhoff A, Inderfurth N, Veuskens T, Schraa G, Blokland M, Kujawa-Roeleveld K, Rijnaarts H 2013 Microbial removal of the pharmaceutical compounds ibuprofen and diclofenac from wastewater. Biomed Res Int 2013:325806. doi:10.1155/2013/325806. 24350260 PMC3852090

[60] Marchlewicz A, Guzik U, Hupert-Kocurek K, Nowak A, Wilczynska S, Wojcieszynska D 2017 Toxicity and biodegradation of ibuprofen by Bacillus thuringiensis B1(2015b). Environ Sci Pollut Res Int 24:7572–7584. doi:10.1007/s11356-017-8372-3. 28116629 PMC5383686

[61] Chen Y, Rosazza JP 1994 Microbial transformation of ibuprofen by a Nocardia species. Appl Environ Microbiol 60:1292–1296. 16349237 10.1128/aem.60.4.1292-1296.1994 PMC201472

[62] Hutt AJ, Kooloobandi A, Hanlon GW 1993 Microbial metabolism of 2-arylpropionic acids: chiral inversion of ibuprofen and 2-phenylpropionic acid. Chirality 5:596–601. doi:10.1002/chir.530050806. 8305287

[63] Kater A, Henke MO, Rubin BK 2007 The role of DNA and actin polymers on the polymer structure and rheology of cystic fibrosis sputum and depolymerization by gelsolin or thymosin beta 4. Ann N Y Acad Sci 1112:140–153. doi:10.1196/annals.1415.006. 17496063

[64] Rubin BK 2007 Mucus structure and properties in cystic fibrosis. Paediatr Respir Rev 8:4–7. doi:10.1016/j.prrv.2007.02.004. 17419972

[65] Ohman DE, Chakrabarty AM 1982 Utilization of human respiratory secretions by mucoid Pseudomonas aeruginosa of cystic fibrosis origin. Infect Immun 37:662–669. 6811437 10.1128/iai.37.2.662-669.1982 PMC347583

[66] Palmer KL, Mashburn LM, Singh PK, Whiteley M 2005 Cystic fibrosis sputum supports growth and cues key aspects of Pseudomonas aeruginosa physiology. J Bacteriol 187:5267–5277. doi:10.1128/JB.187.15.5267-5277.2005. 16030221 PMC1196007

[67] Rice AR, Hamilton MA, Camper AK 2000 Apparent surface associated lag time in growth of primary biofilm cells. Microb Ecol 40:8–15. doi:10.1007/s002480000011. 10977872

[68] Alem MA, Douglas LJ 2004 Effects of aspirin and other nonsteroidal anti-inflammatory drugs on biofilms and planktonic cells of Candida albicans. Antimicrob Agents Chemother 48:41–47. doi:10.1128/AAC.48.1.41-47.2004. 14693516 PMC310207

[69] del Prado G, Ruiz V, Naves P, Rodriguez-Cerrato V, Soriano F, del Carmen Ponte M 2010 Biofilm formation by Streptococcus pneumoniae strains and effects of human serum albumin, ibuprofen, N-acetyl-l-cysteine, amoxicillin, erythromycin, and levofloxacin. Diagn Microbiol Infect Dis 67:311–318. doi:10.1016/j.diagmicrobio.2010.03.016. 20638597

[70] Naves P, del Prado G, Huelves L, Rodriguez-Cerrato V, Ruiz V, Ponte MC, Soriano F 2010 Effects of human serum albumin, ibuprofen and N-acetyl-l-cysteine against biofilm formation by pathogenic Escherichia coli strains. J Hosp Infect 76:165–170. doi:10.1016/j.jhin.2010.05.011. 20615578

[71] Reslinski A, Dabrowiecki S, Glowacka K 2015 The impact of diclofenac and ibuprofen on biofilm formation on the surface of polypropylene mesh. Hernia 19:179–185. doi:10.1007/s10029-013-1200-x. 24366755 PMC4372680

[72] Muller E, Al-Attar J, Wolff AG, Farber BF 1998 Mechanism of salicylate-mediated inhibition of biofilm in Staphylococcus epidermidis. J Infect Dis 177:501–503. doi:10.1086/517386. 9466548

[73] Farber BF, Wolff AG 1992 The use of nonsteroidal antiinflammatory drugs to prevent adherence of Staphylococcus epidermidis to medical polymers. J Infect Dis 166:861–865. doi:10.1093/infdis/166.4.861. 1527423

[74] Demirag MK, Esen S, Zivalioglu M, Leblebicioglu H, Keceligil HT 2007 The effect of aspirin on adherence of slime-producing, coagulase-negative staphylococci to vascular grafts. Ann Vasc Surg 21:464–467. doi:10.1016/j.avsg.2006.06.006. 17628264

[75] Drago L, De Vecchi E, Nicola L, Valli M, Gismondo MR 2002 Effects of subinhibitory concentrations of ibuprofen isobuthanolammonium on virulence factors of uropathogenic Escherichia coli. J Chemother 14:314–315. doi:10.1179/joc.2002.14.3.314. 12120890

[76] Bryers JD, Jarvis RA, Lebo J, Prudencio A, Kyriakides TR, Uhrich K 2006 Biodegradation of poly(anhydride-esters) into non-steroidal anti-inflammatory drugs and their effect on Pseudomonas aeruginosa biofilms in vitro and on the foreign-body response in vivo. Biomaterials 27:5039–5048. doi:10.1016/j.biomaterials.2006.05.034. 16777217 PMC6639030

[77] Ulusoy S, Bosgelmez-Tinaz G 2013 Nonsteroidal anti-inflammatory drugs reduce the production of quorum sensing regulated virulence factors and swarm in motility in human pathogen Pseudomonas aeruginosa [corrected]. Drug Res (Stuttg) 63:409–413. doi:10.1055/s-0033-1343430. 23599038

[78] Oermann CM, Sockrider MM, Konstan MW 1999 The use of anti-inflammatory medications in cystic fibrosis: trends and physician attitudes. Chest 115:1053–1058. doi:10.1378/chest.115.4.1053. 10208207

[79] Onischuk AA, Tolstikova TG, Sorokina IV, Zhukova NA, Baklanov AM, Karasev VV, Borovkova OV, Dultseva GG, Boldyrev VV, Fomin VM 2009 Analgesic effect from ibuprofen nanoparticles inhaled by male mice. J Aerosol Med Pulm Drug Deliv 22:245–253. doi:10.1089/jamp.2008.0721. 19466908

[80] Onischuk AA, Tolstikova TG, Sorokina IV, Zhukova NA, Baklanov AM, Karasev VV, Dultseva GG, Boldyrev VV, Fomin VM 2008 Anti-inflammatory effect from indomethacin nanoparticles inhaled by male mice. J Aerosol Med Pulm Drug Deliv 21:231–243. doi:10.1089/jamp.2007.0672. 18627274

[81] Patton JS 1996 Mechanisms of macromolecule absorption by the lungs. Adv Drug Deliv Rev 19:3–36. doi:10.1016/0169-409X(95)00113-L.

[82] Patton JS, Fishburn CS, Weers JG 2004 The lungs as a portal of entry for systemic drug delivery. Proc Am Thorac Soc 1:338–344. doi:10.1513/pats.200409-049TA. 16113455

[83] Tang BC, Dawson M, Lai SK, Wang YY, Suk JS, Yang M, Zeitlin P, Boyle MP, Fu J, Hanes J 2009 Biodegradable polymer nanoparticles that rapidly penetrate the human mucus barrier. Proc Natl Acad Sci U S A 106:19268–19273. doi:10.1073/pnas.0905998106. 19901335 PMC2780804

[84] van Heeckeren AM, Tscheikuna J, Walenga RW, Konstan MW, Davis PB, Erokwu B, Haxhiu MA, Ferkol TW 2000 Effect of Pseudomonas infection on weight loss, lung mechanics, and cytokines in mice. Am J Respir Crit Care Med 161:271–279. doi:10.1164/ajrccm.161.1.9903019. 10619831

[85] Darling P, Chan M, Cox AD, Sokol PA 1998 Siderophore production by cystic fibrosis isolates of Burkholderia cepacia. Infect Immun 66:874–877. 9453660 10.1128/iai.66.2.874-877.1998 PMC107988

[86] Reik R, Spilker T, Lipuma JJ 2005 Distribution of Burkholderia cepacia complex species among isolates recovered from persons with or without cystic fibrosis. J Clin Microbiol 43:2926–2928. doi:10.1128/JCM.43.6.2926-2928.2005. 15956421 PMC1151955

[87] Spilker T, Uluer AZ, Marty FM, Yeh WW, Levison JH, Vandamme P, Lipuma JJ 2008 Recovery of Herbaspirillum species from persons with cystic fibrosis. J Clin Microbiol 46:2774–2777. doi:10.1128/JCM.00460-08. 18524958 PMC2519502

[88] Heydorn A, Nielsen AT, Hentzer M, Sternberg C, Givskov M, Ersboll BK, Molin S 2000 Quantification of biofilm structures by the novel computer program COMSTAT. Microbiology 146(Pt 10):2395–2407. 11021916 10.1099/00221287-146-10-2395

[89] Sternberg C, Tolker-Nielsen T 2006 Growing and analyzing biofilms in flow cells. Curr Protoc Microbiol Chapter 1:Unit 1B.2. doi:10.1002/9780471729259.mc01b02s00. 18770573

[90] Schindelin J, Arganda-Carreras I, Frise E, Kaynig V, Longair M, Pietzsch T, Preibisch S, Rueden C, Saalfeld S, Schmid B, Tinevez JY, White DJ, Hartenstein V, Eliceiri K, Tomancak P, Cardona A 2012 Fiji: an open-source platform for biological-image analysis. Nat Methods 9:676–682. doi:10.1038/nmeth.2019. 22743772 PMC3855844

[91] Hutchison JB, Rodesney CA, Kaushik KS, Le HH, Hurwitz DA, Irie Y, Gordon VD 2014 Single-cell control of initial spatial structure in biofilm development using laser trapping. Langmuir 30:4522–4530. doi:10.1021/la500128y. 24684606

[92] Kragh KN, Hutchison JB, Melaugh G, Rodesney C, Roberts AE, Irie Y, Jensen PO, Diggle SP, Allen RJ, Gordon V, Bjarnsholt T 2016 Role of multicellular aggregates in biofilm formation. mBio 7:e00237-16. doi:10.1128/mBio.00237-16. 27006463 PMC4807362

[93] Shah PN, Lin LY, Smolen JA, Tagaev JA, Gunsten SP, Han DS, Heo GS, Li Y, Zhang F, Zhang S, Wright BD, Panzner MJ, Youngs WJ, Brody SL, Wooley KL, Cannon CL 2013 Synthesis, characterization, and in vivo efficacy of shell cross-linked nanoparticle formulations carrying silver antimicrobials as aerosolized therapeutics. ACS Nano 7:4977–4987. doi:10.1021/nn400322f. 23718195 PMC4287418

[94] Geller DE, Rosenfeld M, Waltz DA, Wilmott RW, AeroDose TOBI Study Group. 2003 Efficiency of pulmonary administration of tobramycin solution for inhalation in cystic fibrosis using an improved drug delivery system. Chest 123:28–36. doi:10.1378/chest.123.1.28. 12527599

[95] Kovach K, Davis-Fields M, Irie Y, Jain K, Doorwar S, Vuong K, Dhamani N, Mohanty K, Touhami A, Gordon VD 2017 Evolutionary adaptations of biofilms infecting cystic fibrosis lungs promote mechanical toughness by adjusting polysaccharide production. NPJ Biofilms Microbiomes 3:1. doi:10.1038/s41522-016-0007-9. 28649402 PMC5445605

