# Supplementary material for: Antimicrobial Activity of Ibuprofen against Cystic Fibrosis-Associated Gram-Negative Pathogens
Source: Antimicrob Agents Chemother. 2018 Feb 23;62(3):e01574-17. doi: 10.1128/AAC.01574-17 (PMC5826130; doi:10.1128/AAC.01574-17)
Supplement: Supplemental material [file supp_62_3_e01574-17__index.html]

Supplemental material 

# Antimicrobial Activity of Ibuprofen against Cystic Fibrosis-Associated Gram-Negative Pathogens

## Supplemental material

- Supplemental file 1 -

  Supplemental Figure S1

  PDF, 200K
