# Supplementary material for: Antimicrobial Activity of Ibuprofen against Cystic Fibrosis-Associated Gram-Negative Pathogens
Source: Antimicrob Agents Chemother. 2018 Feb 23;62(3):e01574-17. doi: 10.1128/AAC.01574-17 (PMC5826130; doi:10.1128/AAC.01574-17)
Supplement: Supplemental material [file AAC.01574-17_zac003186949s1.pdf]

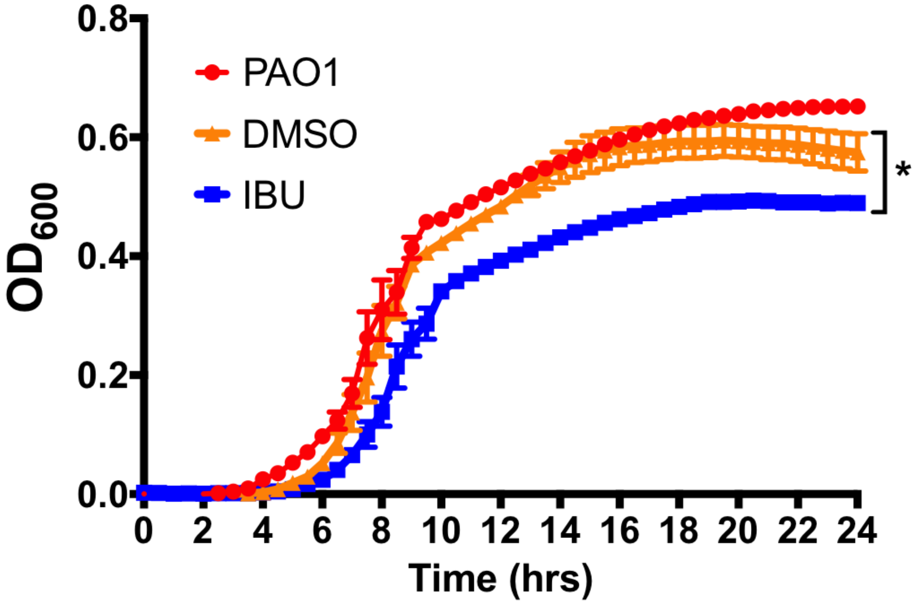

Supplementary Figure – Ibuprofen reduces the bacterial growth rate of P. aeruginosa strain PAO1 cultured rich media. PAO1 was grown at 37ºC in MH broth in the presence of ibuprofen (IBU) (100 µg/mL) over a 24-hour period and OD_{600} was determined at 30 minute intervals. Controls included un-inoculated growth media (not shown), growth medium with bacteria alone, and growth medium containing DMSO (5% v/v) without ibuprofen (0 µg/mL) inoculated with bacteria. Each dataset represents the mean of 2 independent experiments, each with 4 technical replicates (total 8 replicates per treatment) and data are presented as mean ± standard error. * indicates $P < 0.05$, where a paired t-test was used to analyze the differences in end-point OD_{600} for the growth curve.
